# Recent Advances of *N*-2,2,2-Trifluoroethylisatin Ketimines in Organic Synthesis

**DOI:** 10.3390/molecules28072990

**Published:** 2023-03-27

**Authors:** Yuting Liu, Lijie Wang, Donglai Ma, Yongxing Song

**Affiliations:** 1 College of Pharmacy, Hebei University of Chinese Medicine, Shijiazhuang 050200, China; 2 Hebei Technology Innovation Center of TCM Formula Preparations, Hebei University of Chinese Medicine, Shijiazhuang 050200, China; 3 Hebei Technological Innovation Center of Chiral Medicine, Hebei Chemical & Pharmaceutical College, Shijiazhuang 050026, China

**Keywords:** *N*-2,2,2-trifluoroethylisatin ketimine, trifluoromethyl, cycloaddition, organic synthesis, cascade reaction

## Abstract

The special properties of fluorine atoms and fluorine-containing groups have led to an increasing number of applications for fluorine-containing organic compounds, which are also extremely widely used in the field of new drug development. Unfortunately, naturally fluorinated organics are rare in nature, so the selective introduction of fluorine atoms or fluorine-containing groups into organic molecules is very important for pharmaceutical/synthetic chemists. *N*-2,2,2-trifluoroethylisatin ketimines have received the attention of many chemists since they were first developed as fluorine-containing synthons in 2015. This paper reviews the organic synthesis reactions in which trifluoroethyl isatin ketimine has been involved in recent years, focusing on the types of reactions and the stereoselectivity of products, and also provides a prospect of its application in this field.

## 1. Introduction

**Figure 1:**
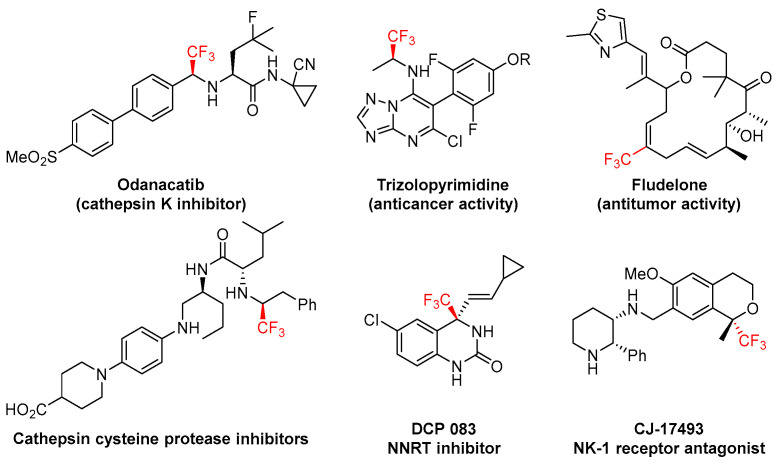
Selected biologically active molecules bearing a CF_3_.

The special properties of fluorinated compounds make them have important application value in medicinal chemistry, pesticides, functional materials, and other fields [1]. Among these fluorinated compounds, trifluoromethylation products account for a large proportion. Trifluoromethyl (CF_3_) is an important fluorine-containing group. The introduction of this functional group into drug molecules often significantly changes the fat solubility of the parent compound, enhances the metabolic stability of the molecule, and affects its biological activities such as drug absorption, distribution, and donor-receptor interaction [2]. Trifluoromethyl is widely found in a variety of bioactive molecules and lead compound structures, with protease inhibition, anticancer, anti-tumor, anti-HIV, and other activities (Figure 1) [3,4,5,6]. For example, as a drug for the treatment of osteoporosis, odanacatib can effectively and selectively inhibit the activity of cathepsin K [7,8]. Fludelone has the same anti-tumor activity as Epothilone D without trifluoromethyl, and the drug has a longer action time and less toxicity and side effects [9]. CJ-17493, as a new NK-1 receptor antagonist, has become a potential therapeutic drug for the treatment of chemotherapy-induced vomiting, arthritis, migraine, and other diseases [10]. In view of the fact that trifluoromethyl can improve and enhance the activity of drugs, the synthesis of trifluoromethyl compounds is becoming a hot topic for many pharmacologists and chemists.

**Scheme 1:**
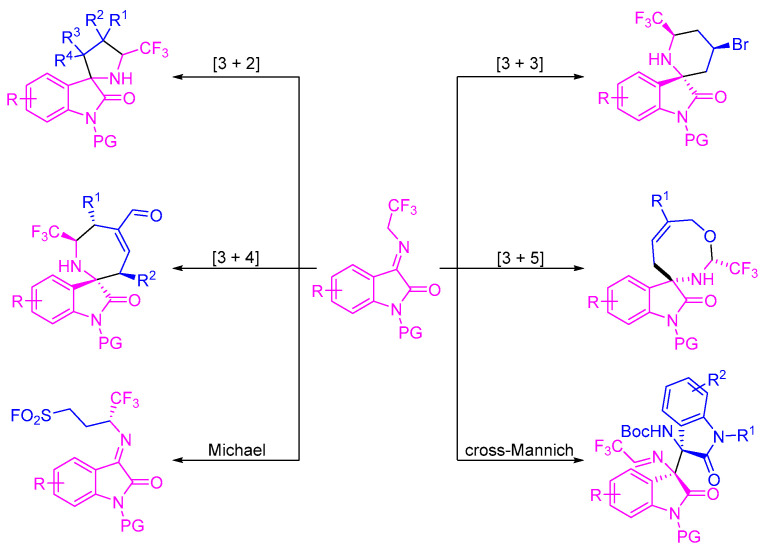
Different types of catalytic reactions involving *N*-2,2,2-trifluoroethylisatin ketimines.

As an important synthetic “building block” containing trifluoromethyl, trifluoroethylamine has made great achievements in the application of organic synthesis [11,12,13,14,15,16]. However, there are few studies on the direct use of trifluoroethylamine in the construction of functionalized α-trifluoromethyl amine compounds. Until 2015, Wang and co-workers synthesized *N*-2,2,2-trifluoroethylisatin ketimine derivatives as 1,3-dipoles for the first time and successfully applied them in an organocatalytic asymmetric [3 + 2] cycloaddition reaction [17]. From then on, the door to the application of trifluoroethyl isatin ketimine in organocatalytic synthesis has been opened. In recent years, with the continuous efforts of organic chemists, many efficient new catalytic systems have been developed, expanding the application range of this type of compound in organic synthesis. In order to summarize the latest research progress on this type of compound, this review describes the application of trifluoroethyl isatin ketimine in organic synthesis based on the stereoselectivity of the products and their reaction types (Scheme 1). This article aims to provide support for the development of trifluoroethyl isatin ketimines in this field by summarizing and analyzing their advantages and disadvantages, reaction mechanisms, and applications in organic synthesis.

## 2. Organocatalytic Reactions Involving *N*-2,2,2-Trifluoroethylisatin Ketimines

As an important synthon of trifluoromethylation, trifluoroethyl isatin ketimines have become attractive partners in organocatalytic reactions in recent years. In order to facilitate combing and better understanding, this review mainly consists of two main parts according to the stereoselectivity of the product, namely the chiral part and the racemic part.

### 2.1. Catalytic Asymmetric Reaction of Trifluoroethyl Isatin Ketimines

#### 2.1.1. Organocatalytic Asymmetric [3 + 2] Cycloaddition Reaction

**Scheme 2:**
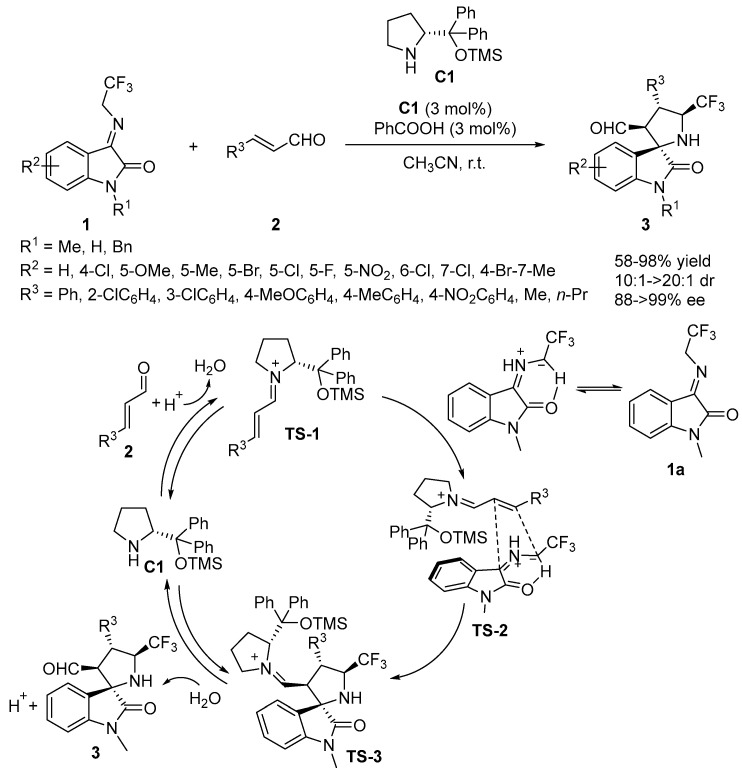
Asymmetric [3 + 2] cycloaddition of trifluoroethyl isatin ketimines to cinnamaldehydes.

In 2015, Wang and co-workers synthesized *N*-2,2,2-trifluoroethylisatin ketimines for the first time and used it as a cascade reaction reagent to undergo an asymmetric [3 + 2] cycloaddition reaction with cinnamaldehyde under the catalysis of prolinol silyl ether (Scheme 2) [17]. This reaction is concise and efficient to obtain chiral spirooxindole derivatives **3** with excellent diastereoselectivities (10:1–>20:1 dr) and enantioselectivities (88–>99% ee) in moderate to excellent yields (58–98%). In addition, researchers proposed a possible transition state model for the reaction based on the absolute configuration of the product. As shown in Scheme 2, prolinol silyl ether reacts with cinnamaldehyde to generate the iminium ion intermediate **TS-1**. Due to the steric hindrance of the aryl group on the *Re*-face, the cycloaddition reaction takes place on the *Si*-face of oxindole-derived azomethine ylides. Then, the intermediate **TS-3** is hydrolyzed to release the product from the catalytic cycle and regenerate the catalyst.

**Scheme 3:**
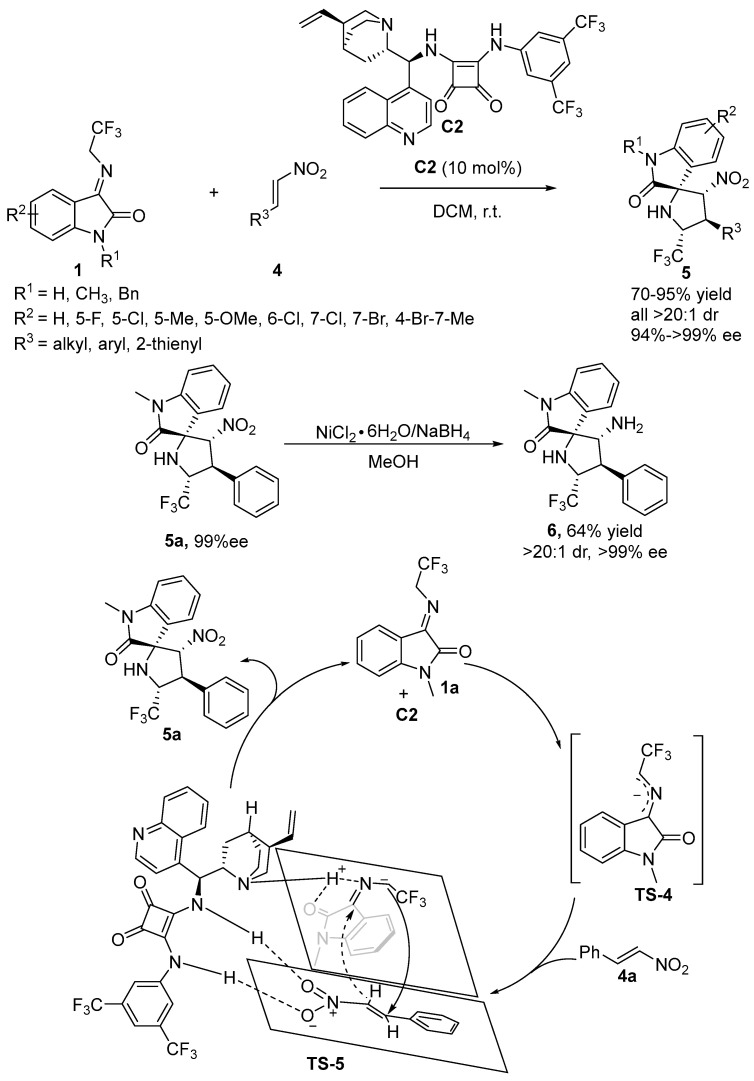
Squaramide-catalyzed [3 + 2] cycloaddition of nitroalkenes with *N*-2,2,2-trifluoroethylisatin ketimines.

In the same year, Wang and co-workers again reported a [3 + 2] cycloaddition reaction for the synthesis of 5′-trifluoromethyl-spiro[pyrrolidin-3,2′-oxindoles] **5** from nitroolefins **4** and *N*-2,2,2-trifluoroethylisatin ketimines **1** catalyzed by squaramide **C2** (Scheme 3) [18]. A series of chiral fluorospiroindole derivatives were synthesized by this reaction in high yields (70–95%) with excellent diastereo- and enantioselectivities (all >20:1 dr and 94–>99% ee). In order to increase the practicability of the product, the adduct **5a** was reduced to its amino derivative **6** in a 64% yield by using NiCl_2_·6H_2_O and NaBH_4_ in methanol, and its stereoselectivity was maintained. In order to better clarify the reaction, the researchers gave a possible reaction mechanism. As shown in Scheme 3, the squaramide catalyst plays a dual activation role. The ketamine **1a** combines with the tertiary amine moiety of the catalyst to form a five-membered ring, and the nitroalkene **4a** is simultaneously fixed and activated by the N-H bond of the squaramide. Subsequently, the product **5a** with a specific configuration was formed after two *Re*-face attacks, and the catalytic cycle was completed.

**Scheme 4:**
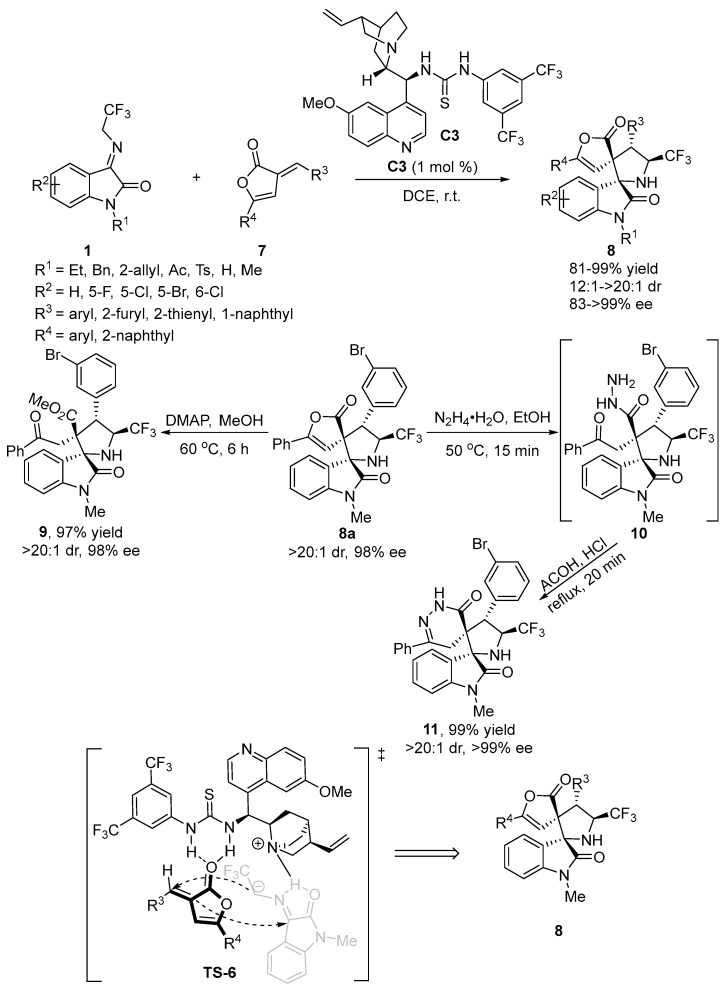
Organocatalytic asymmetric [3 + 2] cycloaddition of *N*-2,2,2-trifluoroethylisatin ketimines with 3-alkenyl-5-arylfuran-2(3*H*)-ones.

An efficient, highly asymmetric [3 + 2] cycloaddition reaction catalyzed by the thiourea-tertiary amine catalyst **C3** for the synthesis of spiro[pyrrolidin-3,2′-oxindoles] **8** was developed by the Yuan group in 2016 (Scheme 4) [19]. This cascade reaction proceeded well at low catalyst loading (1 mol%) with a broad substrate scope, furnishing the desired products in high yields (81–99%) with excellent stereoselectivities (12:1–>20:1 dr and 83–>99% ee) under mild conditions. The practicability of this process was further verified by preparative-scale experiments. Regardless of the catalyst loading of 5 mol% or 1 mol%, the gram-scale reaction can achieve satisfactory results. Subsequently, product **8a** was converted to other spirocyclic oxindoles by treatment with different reagents. Treatment of product **8a** with DMAP in methanol afforded the ring-opened esterified derivative **9** with excellent stereoselectivity (>20:1 dr, 98% ee) in 97% yield. Furthermore, product **8a** could also be converted by hydrazinolysis to the intermediate hydrazide **10**, which was directly treated with a mixture of HCl/AcOH (*v*/*v* 4:1) to give the spirocyclic compound **11** with excellent diastereoselectivity (>20:1 dr) and enantioselectivity (>99% ee) in 99% yield. Based on the experimental data and the absolute configuration of the product, a transition state model of the reaction was proposed, which further explained that the reaction was catalyzed by a tertiary amine-thiourea bifunctional activation mode to achieve substrate activation and stereoselectivity control.

**Scheme 5:**
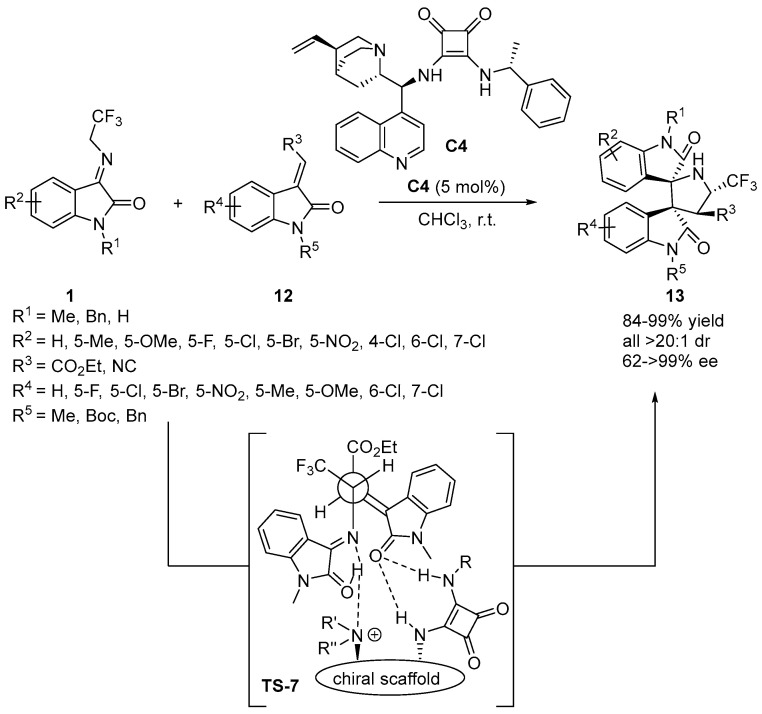
Asymmetric [3 + 2] cycloaddition reaction of *N*-2,2,2-trifluoroethylisatin ketimines with methyleneindolinones.

In 2016, Lu and co-workers disclosed a highly efficient asymmetric [3 + 2] cycloaddition reaction of *N*-2,2,2-trifluoroethylisatin ketimines **1** with methyleneindolinones **12** catalyzed by a bifunctional squaramide-tertiary amine catalyst **C4** (Scheme 5) [20]. This method can synthesize a series of potentially biologically active trifluoromethyl-containing spirooxindole derivatives **13** in excellent yields (84–99%) and stereoselectivities (all >20:1 dr and 62–>99% ee). In addition, the researchers also provided the catalytic reaction model. The squaramide catalyst plays a double activation role, which is similar to the process described in Scheme 3, so it will not be described too much here.

**Scheme 6:**
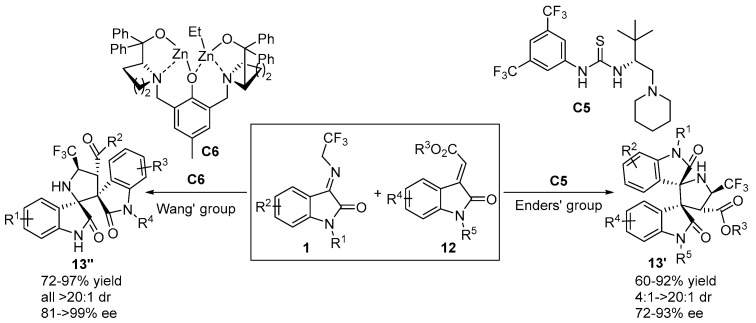
Organocatalytic asymmetric [3 + 2] cycloaddition reaction of *N*-2,2,2-trifluoroethylisatin ketimines with methyleneindolinones.

Subsequently, Enders’ group and Wang’s group reported the asymmetric [3 + 2] cycloaddition reaction of *N*-2,2,2-trifluoroethylisatin ketimines **1** with methyleneindolinones **12** in 2017 and 2020, respectively, using different organic catalysts (Scheme 6) [21,22]. Whether it is a bifunctional thiourea catalyst or an acid-base synergistic catalyst, both of them can efficiently synthesize a series of potentially biologically active trifluoromethyl-containing bispiro indolinone derivatives, which provides a new method for expanding the construction of complex chiral pyrrolidine bispirooxindole skeletons.

**Scheme 7:**
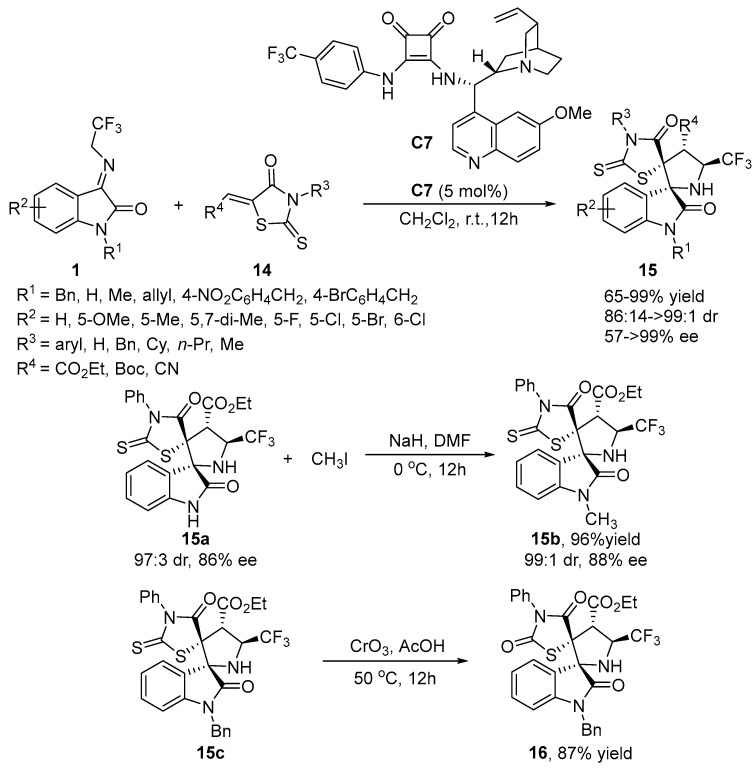
Asymmetric domino Michael/Mannich [3 + 2] cycloaddition reaction between rhodanine derivatives and *N*-2,2,2-trifluoroethylisatin ketimines.

An efficient asymmetric [3 + 2] cycloaddition reaction of *N*-2,2,2-trifluoroethylisatin ketimines **1** with rhodamine derivatives **14** was described by the Du group in 2018 (Scheme 7) [23]. This asymmetric reaction proceeded well with 5 mol% bifunctional cinchona-derived squaramide catalyst **C7** to give CF_3_-containing bispiro heterocyclic compounds **15** in high yields (65–99%) with good diastereo- and enantioselectivities (86:14–>99:1 dr and 57–>99% ee). Based on the experimental results and the absolute configuration of the products, the researchers proposed a possible transition state model for this catalytic cycle. Furthermore, the feasibility of this method was proven by the preparation of the gram scale. At the same time, the practicability of this method was proven by the derivative transformation reaction of the product. Treatment of product **15a** with CH_3_I and NaH in DMF afforded its methylated derivative **15b** a 96% yield while maintaining its stereoselectivity. Treatment of the heterocyclic product **15c** with CrO_3_ in AcOH can readily oxidize its thiocarbonyl group to an oxycarbonyl group.

**Scheme 8:**
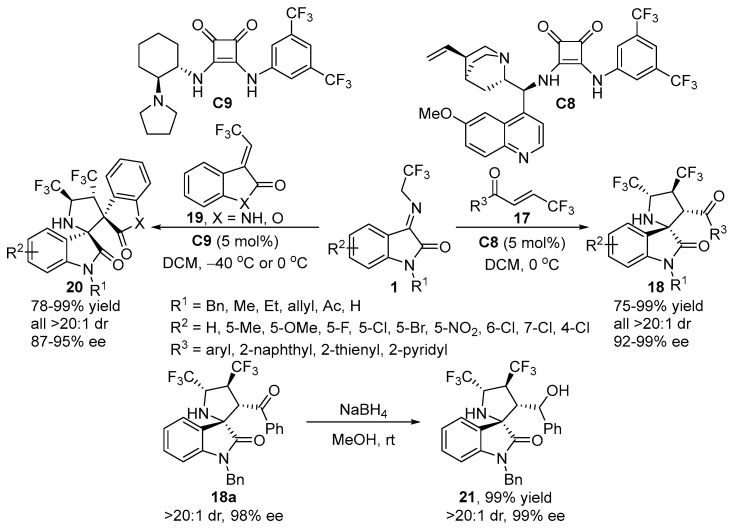
Organocatalytic asymmetric [3 + 2] cycloaddition of *N*-2,2,2-trifluoroethylisatin ketimines with β-trifluoromethyl electron-deficient alkenes.

In 2018, Yuan and co-workers presented bifunctional squaramide-catalyzed [3 + 2] cycloaddition of *N*-2,2,2-trifluoroethylisatin ketimines with β-trifluoromethyl electron-deficient alkenes (Scheme 8) [24]. Under the catalysis of cinchona base-derived squaramide, the vicinally bis(trifluoromethyl)-substituted 3,2′-pyrrolidinyl spirooxindoles with excellent stereoselectivities (all >20:1 dr and 92–>99% ee) can be obtained in good yields (75–99%) using β-trifluoromethyl enones as electron-deficient alkenes. Notably, under the catalysis of cyclohexanediamine-derived squaramide, a series of vicinally bis(trifluoromethyl)-substituted 3,2′-pyrrolidinyl spirooxindoles with four contiguous stereocenters, including two vicinal spiro-quaternary chiral center atoms, can be obtained in high yields (78–99%) with excellent diastereoselectivities (all >20:1 dr) and enantioselectivities (87–95% ee) by using 3-trifluoroethylidene oxindole and 3-trifluoroethylidene benzofuranone as substrates. In addition, the practicability of this method is proved by the derivation experiment. Treatment of product **18a** with NaBH_4_ in methanol afforded the tertiary alcohol derivative **21** in a 99% yield without loss of its stereoselectivity.

**Scheme 9:**
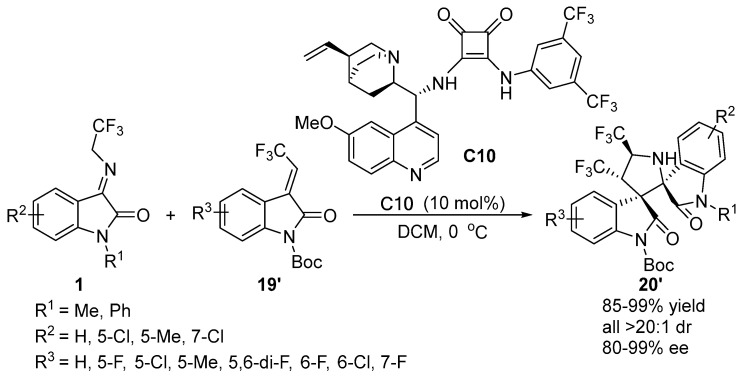
Squaramide-catalyzed asymmetric [3 + 2] cycloaddition of *N*-2,2,2-trifluoroethylisatin ketimines with 3-(trifluoroethylidene)oxindoles.

Subsequently, Lu and co-workers also reported the asymmetric [3 + 2] cycloaddition of *N*-2,2,2-trifluoroethylisatin ketimines **1** to 3-(trifluoroethylidene)oxindoles **19′** catalyzed by squaramide **C10** (Scheme 9) [25]. This process enables the preparation of highly functionalized vicinally bis(trifluoromethyl)-substituted 3,3′-pyrrolidinyl spirooxindoles **20′** in excellent yields (85–99%) with excellent diastereo- and enantioselectivities (all >20:1 dr and 80–99% ee).

**Scheme 10:**
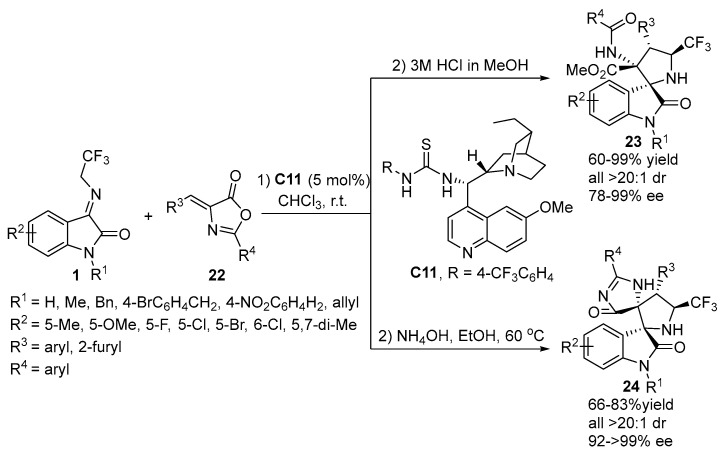
Thiourea-catalyzed domino Michael/Mannich [3 + 2] cycloaddition reaction between *N*-2,2,2-trifluoroethylisatin ketimines and arylidene azlactones.

In 2019, Du and co-workers established an efficient organocatalytic asymmetric [3 + 2] cycloaddition of *N*-2,2,2-trifluoroethylisatin ketimines **1** with arylidene azlactones **22** (Scheme 10) [26]. Under mild conditions, this reaction can easily obtain CF_3_-containing 3,2′-pyrrolidinyl spirooxindole derivatives **23** with excellent diastereoselectivities (all >20:1 dr) and enantioselectivities (78–99% ee) in moderate to excellent yields (60–99%). It is worth noting that CF_3_-containing 3,2′-pyrrolidinyl dispirooxindole derivatives **24** with excellent diastereo- and enantioselectivities (all >20:1 dr and 92–>99% ee), can be obtained in high yields (66–83%) by slightly changing the reaction conditions.

**Scheme 11:**
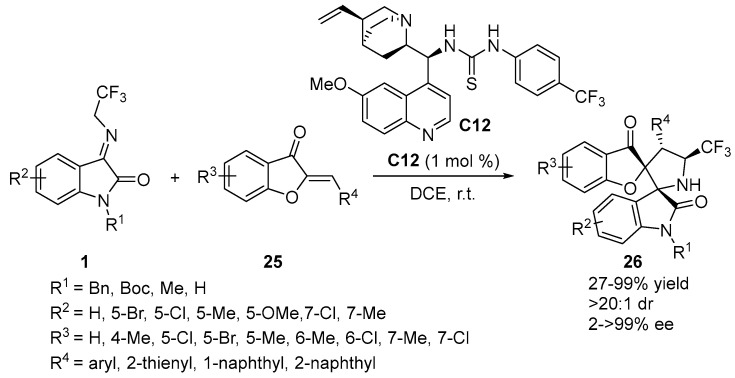
Thiourea-catalyzed 1,3-dipolar cycloaddition reaction of *N*-2,2,2-trifluoroethylisatin ketimines with aurones.

The thiourea-catalyzed asymmetric [3 + 2] cycloaddition reaction of *N*-2,2,2-trifluoroethylisatin ketimines **1** with aurones **25** was developed by the Yan group in 2019 (Scheme 11) [27]. This process enables the construction of a novel spiro [benzofuran-pyrrolidine-oxindole] skeleton with excellent diastereoselectivities (all >20:1 dr) and good enantioselectivities (2–>99% ee) in moderate to excellent yields (27–99%). In addition, researchers described a possible catalytic reaction model. The researchers believe that under the dual activation, the reactants undergo intermolecular Michael addition and intramolecular Mannich reaction to deliver cyclization products.

**Scheme 12:**
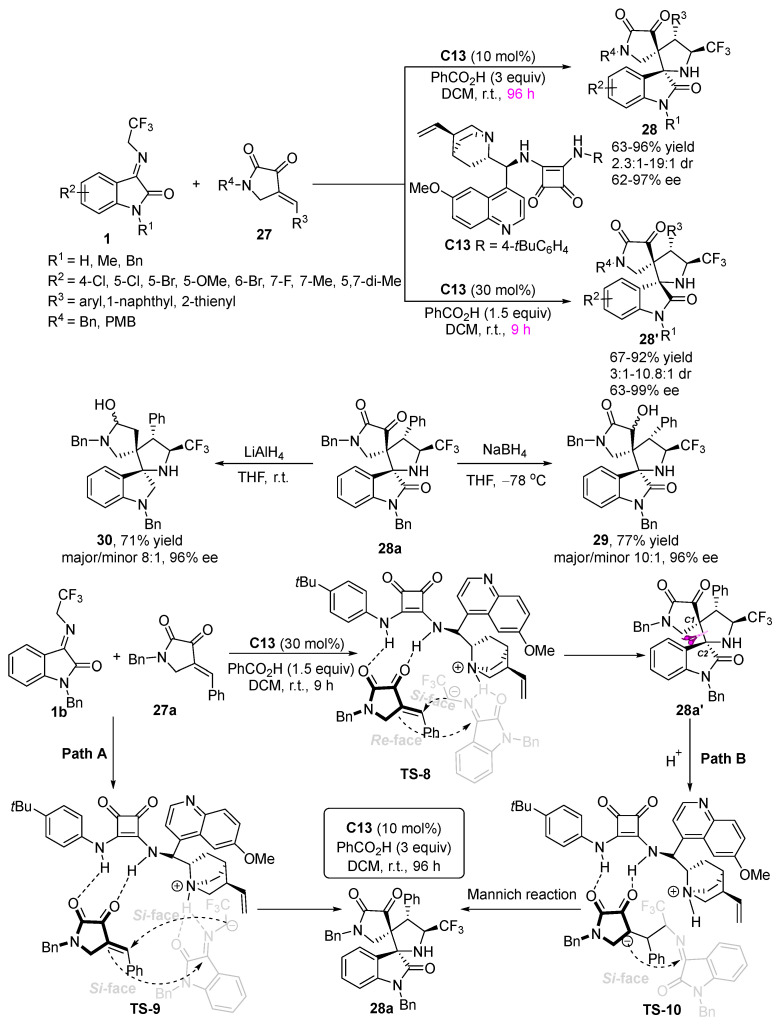
Enantioselective [3 + 2] cycloaddition between *N*-2,2,2-trifluoroethylisatin ketimines and 2,3-dioxopyrrolidines.

In 2019, Jiang and co-workers disclosed a highly asymmetric [3 + 2] cycloaddition between *N*-2,2,2-trifluoroethylisatin ketimines **1** and 2,3-dioxopyrrolidines **27** (Scheme 12) [28]. Under optimal conditions, all reactions proceeded smoothly and afforded a series of chiral spirobipyrrolidine derivatives **28** with two adjacent spiro-quaternary carbon centers in moderate to excellent yields (63–96%) with good diastereoselectivities (2.3:1–19:1 dr) and enantioselectivities (62–97% ee). The practicality of this catalytic reaction was demonstrated by gram-scale and derivatization experiments. It is worth noting that this method can obtain single-configuration diastereoisomers by controlling the reaction time under single catalytic conditions. The researchers thoroughly studied the diastereoselective conversion mechanism through controlled experiments. The squaramide activates pyrrolidine **27a** and indoleketimine **1b** through dual activation modes and controls the stereoselectivity of the Michael(*Si*-face)/Mannich(*Re*-face) cascade reaction (**TS-8**) to obtain compound **28a′**. Meanwhile, equivalent diastereomer **28a** (Path A) can also be obtained by Michael(*Si*-face)/Mannich(*Si*-face) cascade reaction (**TS-9**). If the reaction time is prolonged, the carbon-carbon bond between adjacent spiro-quaternary carbons in **28a′** will be broken under acidic conditions, and the intramolecular Mannich reaction (*Si*-face) will occur again to convert to **28a** (**TS-3**, Path B).

**Scheme 13:**
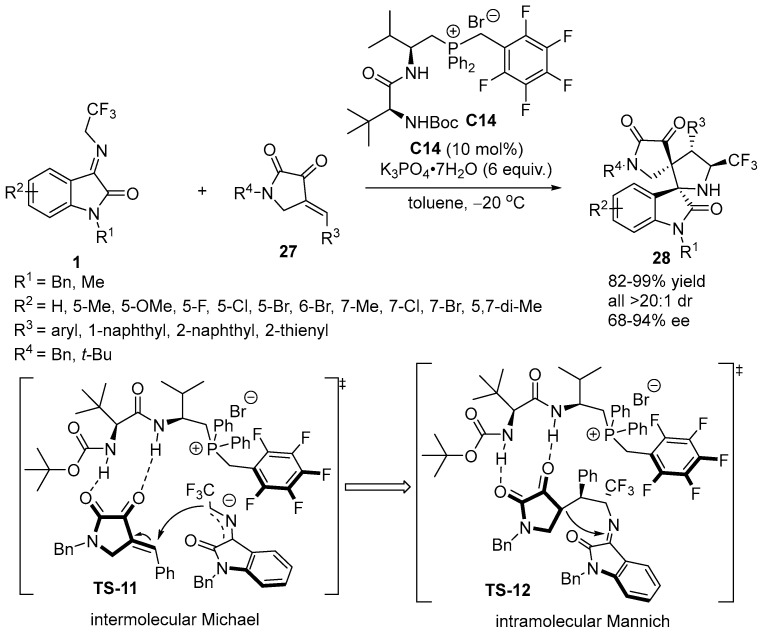
Phosphonium salt-catalyzed [3 + 2] cycloaddition between *N*-2,2,2-trifluoroethylisatin ketimines and 2,3-dioxopyrrolidines.

An alternative method for enantioselective synthesis of CF_3_-containing spirooxindoles via [3 + 2] cycloaddition between *N*-2,2,2-trifluoroethylisatin ketimines **1** and 2,3-dioxopyrrolidines **27** was developed by the Wang group in 2020 (Scheme 13) [29]. The researchers developed a class of chiral bifunctional quaternary phosphonium phase transfer catalysts (**C14**) based on chiral dipeptides and successfully applied them to this reaction. Under mild reaction conditions, this reaction can obtain CF_3_-containing 3,2′-pyrrolidinyl spirooxindole derivatives with excellent stereoselectivities (all >20:1 dr and 68–94% ee) in excellent yields (82–99%). In addition, the researchers confirmed through controlled experiments that chiral quaternary phosphonium salts activate substrates and control stereoselectivity through a synergistic ion-pair/H-bonding effect (**TS-11**, **TS-12**).

**Scheme 14:**
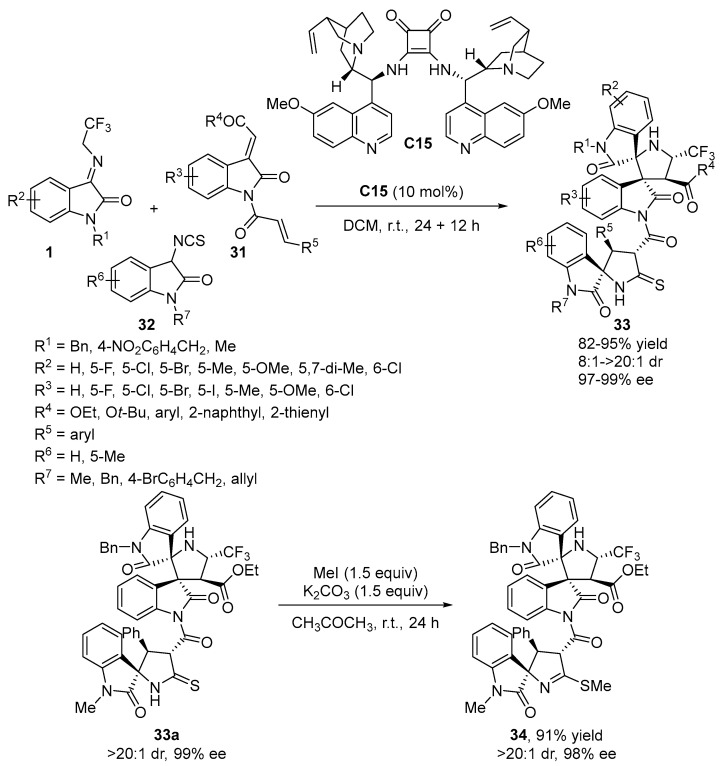
Bifunctional squaramide-catalyzed one-pot three-component Michael/Mannich―Michael/cyclization sequential cascade reactions.

In 2019, Du and co-workers synthesized structurally novel cinnamoyl-3-ylideneoxindoles **31** and successfully applied them in a one-pot, three-component cascade reaction (Scheme 14) [30]. By using a quinine-derived bifunctional squaramide catalyst **C15**, a variety of CF_3_-containing bispirooxindole-spirooxindoles **33** with seven stereocenters were synthesized in high yields (82–95%) with excellent diastereo- and enantioselectivities (8:1–>20:1 dr and 97–99% ee). Furthermore, the comprehensive practicability of this asymmetric catalytic process was further demonstrated by the gram-scale preparation and derivative transformation experiments of product **33a**. Product **33a** was readily reacted with iodomethane under mild conditions to give another family of bispirooxindole-spirooxindole analogues, **34,** with excellent enantioselectivity (98% ee) in 91% yield.

**Scheme 15:**
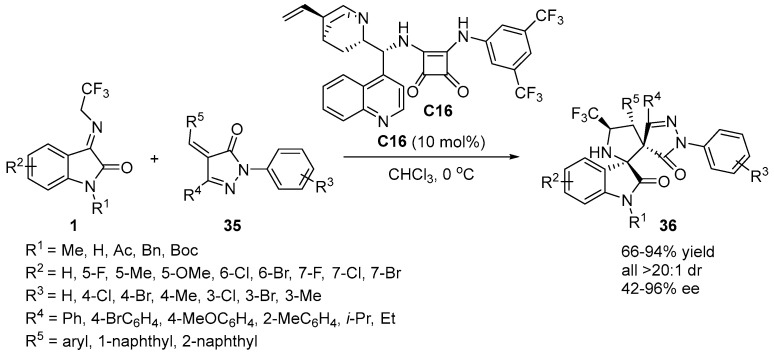
Squaramide-catalyzed asymmetric [3 + 2] cycloaddition of *N*-2,2,2-trifluoroethylisatin ketimines with α,β-unsaturated pyrazolones.

In 2019, Yan and co-workers presented squaramide-catalyzed asymmetric [3 + 2] cycloaddition of *N*-2,2,2-trifluoroethylisatin ketimines **1** with α,β-unsaturated pyrazolones **35** (Scheme 15) [31]. A series of novel spiro-oxindole-pyrrolidine-pyrazolone derivatives **36** with excellent diastereoselectivities (all >20:1 dr) and moderate to excellent enantioselectivities (42–96% ee) were synthesized in high yields (66–94%) under mild conditions. In addition, the researchers also described the dual activation mechanism involved in the reaction in detail.

**Scheme 16:**
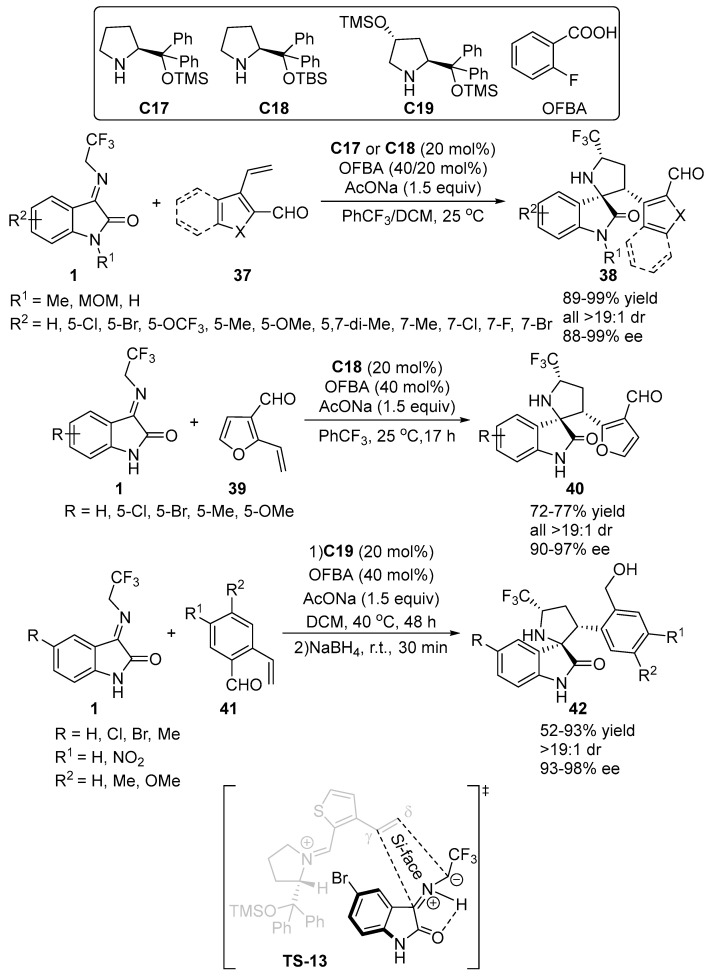
Chiral secondary amine catalyzed asymmetric [3 + 2] cycloaddition reaction of *N*-2,2,2-trifluoroethylisatin ketimines with vinyl substituted aryl aldehydes.

A chiral secondary amine catalyzed asymmetric [3 + 2] cycloaddition reaction of *N*-2,2,2-trifluoroethylisatin ketimines **1** with vinyl substituted aryl aldehydes (**37**, **39**, **41**) was reported by the Chen group in 2019 (Scheme 16) [32]. This reaction has mild conditions and excellent substrate tolerance, making it suitable for trifluoroethylisatin ketimine substrates with different electronegativity and positional substitution. However, for vinyl substituted aromatic aldehydes, the position of the aldehyde group and the vinyl group has a great influence on the reaction activity and stereoselectivity. For the *o*-vinyl benzaldehyde substrate, NO_2_ must be introduced at the 3-position to make the reaction go smoothly. Based on the experimental data and the absolute configuration of the product, the researchers proposed the corresponding catalytic mechanism. The reaction utilizes a chiral prolinol silyl ether catalyst to achieve the activation of vinyl-substituted aromatic aldehydes through a strategy of lowering the lowest unoccupied molecular orbital (LUMO) of polyconjugated imine ions.

**Scheme 17:**
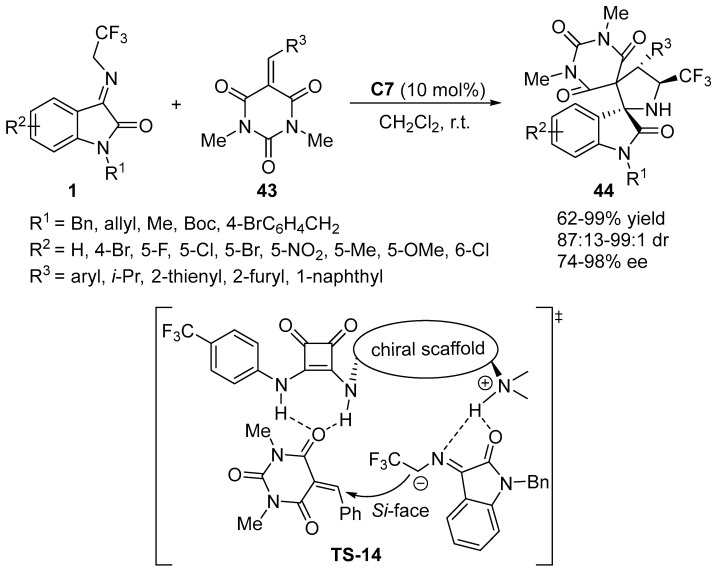
Squaramide-catalyzed asymmetric [3 + 2] cycloaddition of *N*-2,2,2-trifluoroethylisatin ketimines with barbiturate-based olefins.

In the same year, Du and co-workers developed squaramide-catalyzed asymmetric [3 + 2] cycloaddition of *N*-2,2,2-trifluoroethylisatin ketimines **1** with barbiturate-based olefins **43** (Scheme 17) [33]. This reaction proceeds smoothly under mild conditions and has good substrate universality, and spiroheterocyclic derivatives **44** with good diastereoselectivities (87:13–99:1 dr) and enantioselectivities (74–98% ee) can be constructed in moderately excellent yields (62–99%). Meanwhile, the reaction can be carried out smoothly without losing its stereoselectivity by enlarging the scale to 20 times on the basis of the experimental model. Furthermore, the researchers discussed in detail the catalytic model of the dual activation mechanism (**TS-14**) in the reaction based on the experimental data and the absolute configuration of the product.

**Scheme 18:**
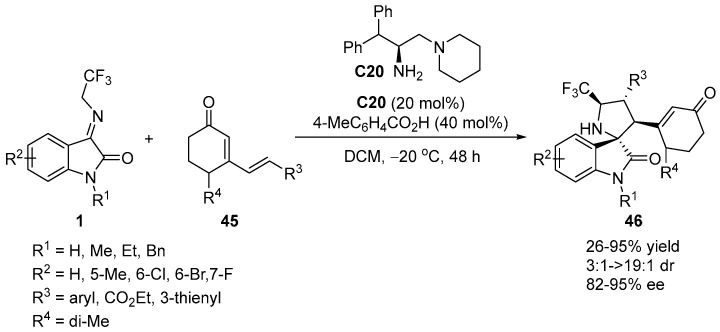
Chiral primary-tertiary amine catalyzed asymmetric [3 + 2] cycloaddition of *N*-2,2,2-trifluoroethylisatin ketimines with cyclic 2,4-dienones.

In 2019, Ye and co-workers reported the use of chiral primary amine **C20** as a catalyst to catalyze the remote regioselective asymmetric [3 + 2] cycloaddition reaction between *N*-2,2,2-trifluoroethyl isatin ketimines **1** and cyclic 2,4-dienones **45** (Scheme 18) [34]. This asymmetric cascade reaction can construct a series of chiral pyrrolidine spirocyclic indole derivatives **46** with good to excellent diastereo- and enantioselectivities (3:1–>19:1 dr and 82–95% ee) in high yields (26–95%).

**Scheme 19:**
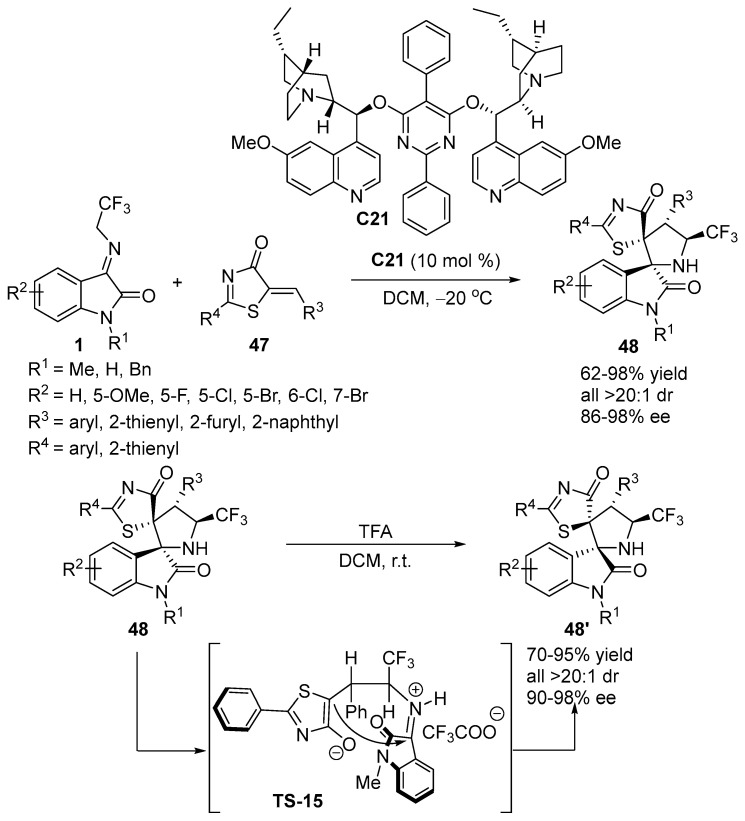
Organocatalytic asymmetric [3 + 2] cycloaddition of *N*-2,2,2-trifluoroethylisatin ketimines with 5-alkenyl thiazolones.

Hydroquinine-derived organocatalysts catalyzed the asymmetric [3 + 2] cycloaddition of *N*-2,2,2-trifluoroethylisatin ketimines **1** with 5-alkenyl thiazolones **47,** as disclosed by the Jiang group in 2020 (Scheme 19) [35]. This method synthesized a series of novel pyrrolidinyl spirooxindole derivatives **48** with excellent stereoselectivities (all >20:1 dr and 86–98% ee) in moderate to excellent yields (62–98%). Interestingly, optically pure products **48** or their racemates can easily undergo configuration conversion under acidic conditions to obtain their diastereoisomers **48′** with high stereoselectivity. The researchers believe that the product **39** undergoes retro-Mannich synthesis under acidic conditions to generate the intermediate **TS-15**, delivering the sterically advantageous **48′** as the only product.

**Scheme 20:**
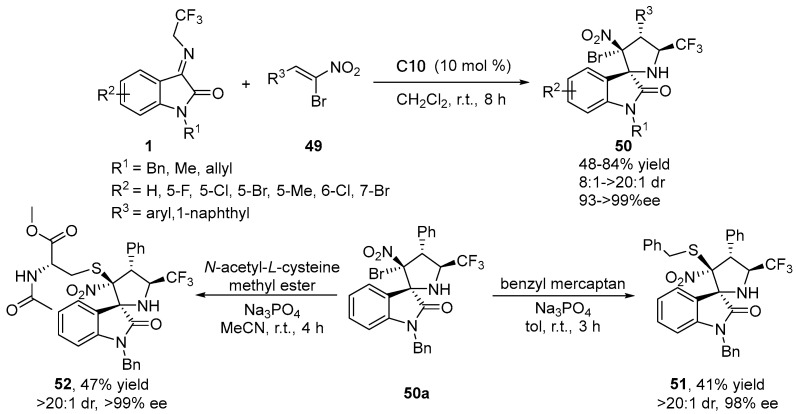
Squaramide-catalyzed asymmetric [3 + 2] cycloaddition of *N*-2,2,2-trifluoroethylisatin ketimines with (*Z*)-α-bromonitroalkenes.

In 2020, Han and co-workers described the squaramide-catalyzed asymmetric [3 + 2] cycloaddition of *N*-2,2,2-trifluoroethylisatin ketimines **1** with (*Z*)-α-bromonitroalkenes **49** (Scheme 20) [36]. This cascade reaction has good substrate tolerance and can construct pyrrolidine-fused spirooxindole derivatives **50** with good diastereoselectivities (8:1–>20:1 dr) and excellent enantioselectivities (93–>99%) in moderate to high yields (48–84%) under optimal conditions. In order to increase the potential drug activity of the product, a derivatization experiment was carried out on it. When product **50a** was treated with the sulfhydryl-containing nucleophiles reagent benzyl mercaptan or *N*-protected cysteine methyl ester, its sulfurized derivatives could be synthesized in moderate yields without loss of its stereoselectivity.

**Scheme 21:**
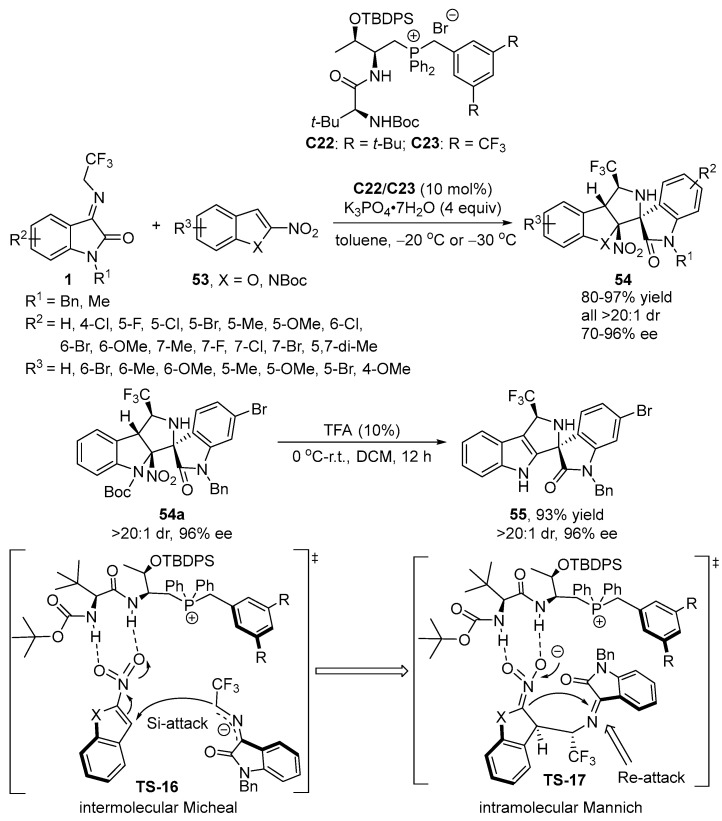
Bifunctinoal phosphonium salt-catalyzed asymmetric [3 + 2] cycloaddition between *N*-2,2,2-trifluoroethylisatin ketimines and 2-nitroindoles or 2-nitrobenzofurans.

A highly efficient asymmetric [3 + 2] annulation reaction of *N*-2,2,2-trifluoroethylisatin ketimines **1** and 2-nitroindoles or 2-nitrobenzofurans was presented by the Wang group in 2021 (Scheme 21) [37]. This reaction has a wide range of substrates, and the CF_3_-containing polycyclic spirooxindole derivatives **54** with excellent diastereoselectivities (all >20:1 dr) and good enantioselectivities (70–96% ee) can be obtained in high yields (80–97%) by dipeptided phosphonium salt catalysis (**C22** or **C23**). Pleasantly, the gram-scale preparation was able to isolate the product with an 87% yield while maintaining its stereoselectivity. Chiral compound **55** was obtained in 93% yield with 96% ee by treating product **54a** with trifluoroacetic acid. This catalytic reaction mechanism is similar to that described in Scheme 13, and the product stereoselectivity is mainly controlled by the ion-pair and H-bonding interactions between chiral phosphonium salts and substrates.

Subsequently, Wang’s group reported a similar reaction for binuclear zinc-catalyzed enantioselective dearomatization [3 + 2] cycloaddition [38]. This strategy provides a series of 2,3-fused dihydrobenzofuran (or dihydrobenzothiophene) derivatives with excellent diastereoselectivities and enantioselectivities in high yields under mild conditions.

**Scheme 22:**
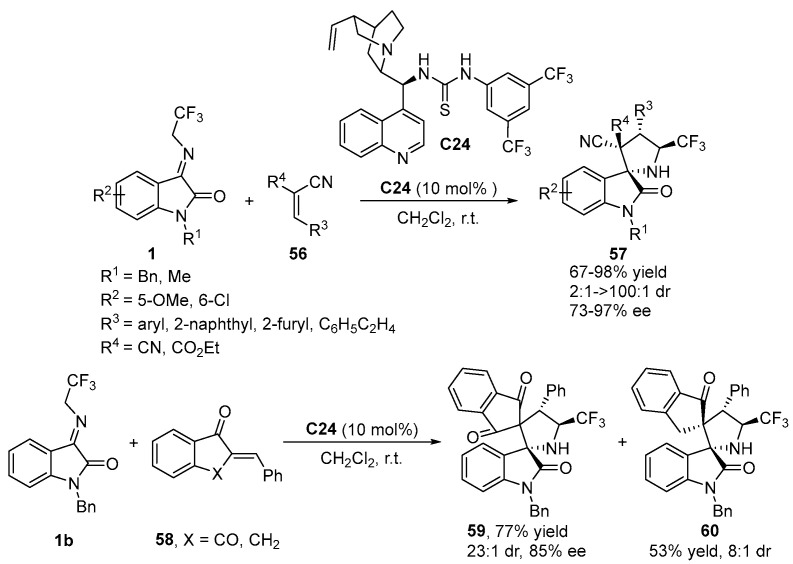
Enantioselective organocatalytic asymmetric [3 + 2] cycloaddition between *N*-2,2,2-trifluoroethylisatin ketimines and benzylidenemalononitriles or benzylidineindanones.

In 2021, Knipe and co-workers established the organocatalytic asymmetric [3 + 2] cycloaddition of *N*-2,2,2-trifluoroethylisatin ketimines **1** with benzylidenemalononitriles **56** catalyzed by a cinchona-derived thiourea catalyst **C24** (Scheme 22) [39]. Under mild conditions, this reaction can provide a series of functionalized spiro-pyrrolidinoxindole derivatives with three to four chiral centers in high yields (67–98%) with good diastereo- and enantioselectivities (2:1–>100:1 dr and 73–97% ee). In addition, the researchers also tried the asymmetric cycloaddition reaction of isatin-derived ketimines **1b** and benzylidene-indanediones **58** under the same reaction conditions. The experimental results showed that no matter whether indenedione or indanone was used as the substrate, the bisspirooxindole derivative could be constructed with moderate yield and diastereoselectivity. Unfortunately, the enantioselectivity of indanone-based products has not been determined.

**Scheme 23:**
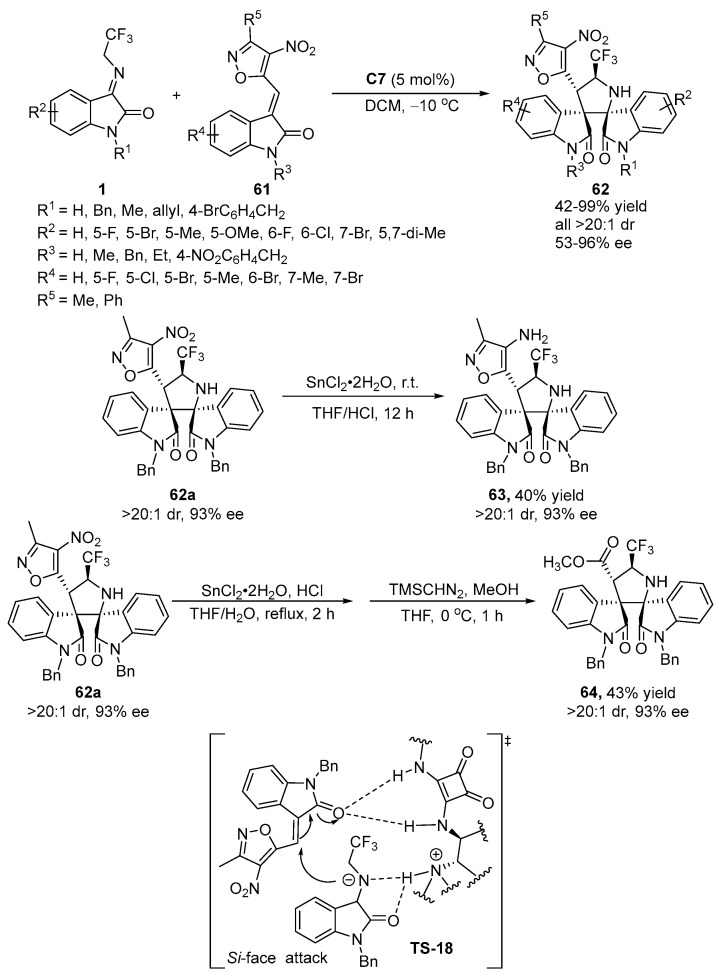
Squaramide-catalysed asymmetric [3 + 2] cycloaddition between *N*-2,2,2-trifluoroethylisatin ketimines and 3-methyl-4-nitro-5-isatylidenyl-isoxazoles.

An efficient and practical squaramide-catalyzed asymmetric domino Micheal/Mannich [3 + 2] annulation reaction of *N*-2,2,2-trifluoroethylisatin ketimines **1** and 3-methyl-4-nitro-5-isatylidenyl-isoxazoles **61** was reported by the Du group in 2022 (Scheme 23) [40]. This asymmetric reaction can obtain a series of CF_3_-containing 3,2′-pyrrolidinyl dispirooxindole derivatives **62** with excellent diastereoselectivities (all >20:1 dr) and good enantioselectivities (53–96% ee) in moderate to excellent yields (42–99%). The practicability of the asymmetric catalytic reaction was proved by the preparation experiment on the gram scale. Moreover, the comprehensive applicability of this method was further proved by the derivative transformation experiment of the product. The nitro group was selectively reduced with tin chloride in a THF/HCl mixed solution at room temperature, and its amine derivative **63** was obtained with a 40% yield. However, by slightly changing the reaction temperature and reagent, the ring-opening product **64** can be obtained with a yield of 43% by a two-step reaction, and its stereoselectivity can be maintained. The researchers detailed the dual activation of the catalyst based on the experimental results and the absolute configuration of the products (**TS-18**).

#### 2.1.2. Organocatalytic Asymmetric [3 + 4] Cycloaddition Reaction

**Scheme 24:**
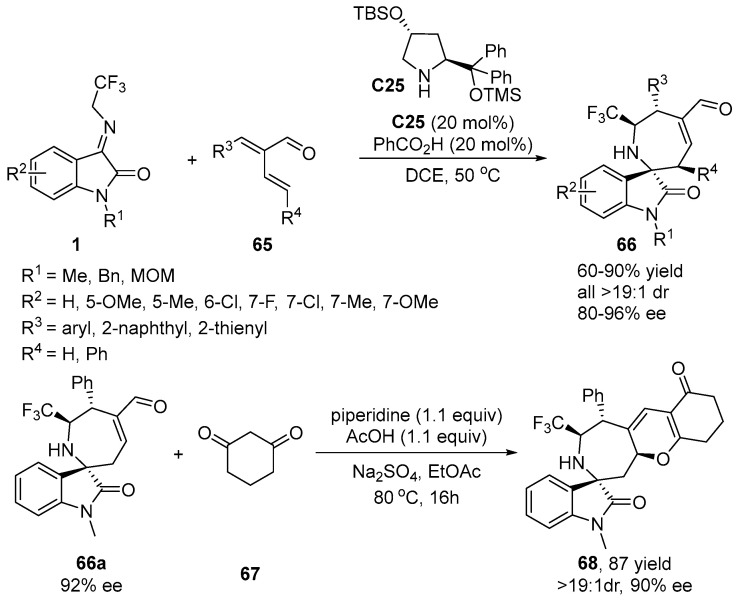
Chiral secondary amine catalyzed asymmetric [3 + 4] cycloaddition between *N*-2,2,2-trifluoroethylisatin ketimines and α-vinylenals.

In 2021, Chen and co-workers developed a highly asymmetric [3 + 4] cycloaddition reaction of *N*-2,2,2-trifluoroethylisatin ketimines **1** and α-vinylenals **65** catalyzed by prolinol silyl ethers **C25** (Scheme 24) [41]. Under optimal conditions, a series of CF_3_-containing spirooxindole derivatives **66** incorporating an azepane motif were synthesized with excellent stereoselectivities (all >19:1 dr and 80–96% ee) and high yields (60–90%). In addition, the α,β-unsaturated aldehyde moiety of the cyclization product **66a** can undergo a [3 + 3] cycloaddition reaction with cyclohexane-1,3-dione **67** under mild conditions and construct a more complex product **68** in 87% yield without losing its stereoselectivity.

#### 2.1.3. Catalytic Asymmetric Reaction of Trifluoroethylisatin Ketimines as Nucleophilic Reagent

**Scheme 25:**
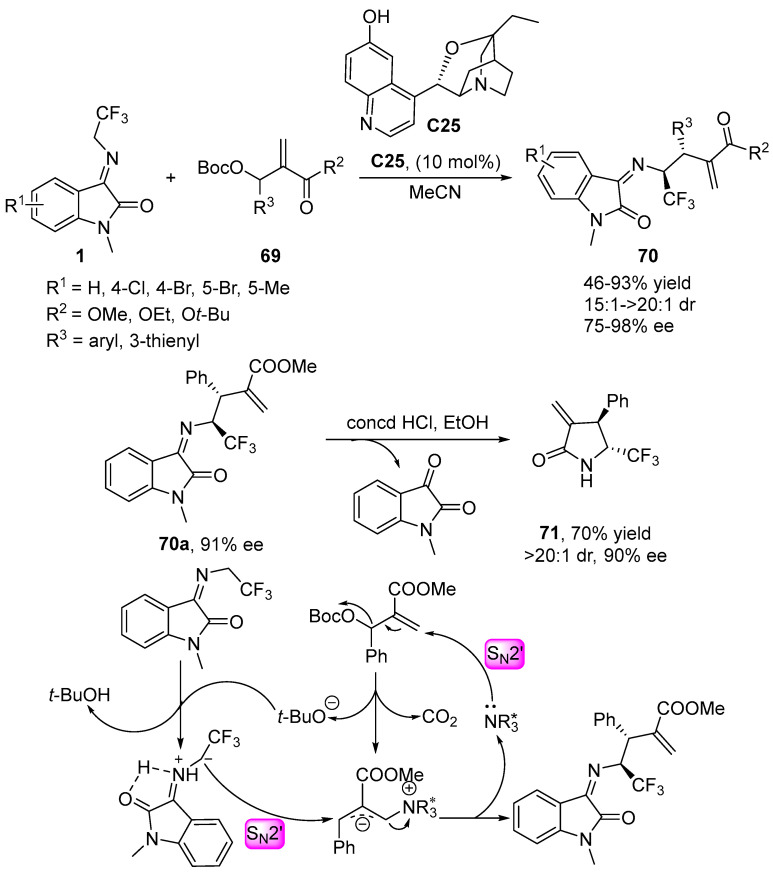
Catalyzed asymmetric S_N_2′-S_N_2′ reaction between *N*-2,2,2-trifluoroethylisatin ketimines and MBH carbonates.

A highly efficient asymmetric S_N_2′-S_N_2′ reaction between *N*-2,2,2-trifluoroethylisatin ketimines **1** and Morita-Baylis-Hillman (MBH) carbonates **69** was disclosed by the Wang group in 2016 (Scheme 25) [42]. In this reaction, a series of chiral α-trifluoromethylamines **70** with good diastereoselectivities (15:1–>20:1 dr) and enantioselectivities (75–98% ee) can be synthesized with moderate to excellent yields (46–93%) by using the catalysts derived from cinchona base. In the mixed solution of concentrated HCl/EtOH, the adduct **70a** can easily remove the *N*-methyl isatin group. The remaining product fraction undergoes self-cyclization and conversion to the pharmacophore α-methylenelactams **71**. Researchers proposed a possible mechanism model for the catalytic reaction based on the absolute configuration of the product. *β*-ICD acts as a Lewis base chiral catalyst to attack MBH carbonate through the S_N_2′ process to remove a molecule of CO_2_ and a tert-butanol anion. Isatinketimine was deprotonated and activated by the tert-butanol anion, and it takes place in another S_N_2′ reaction as a nucleophilic reagent.

**Scheme 26:**
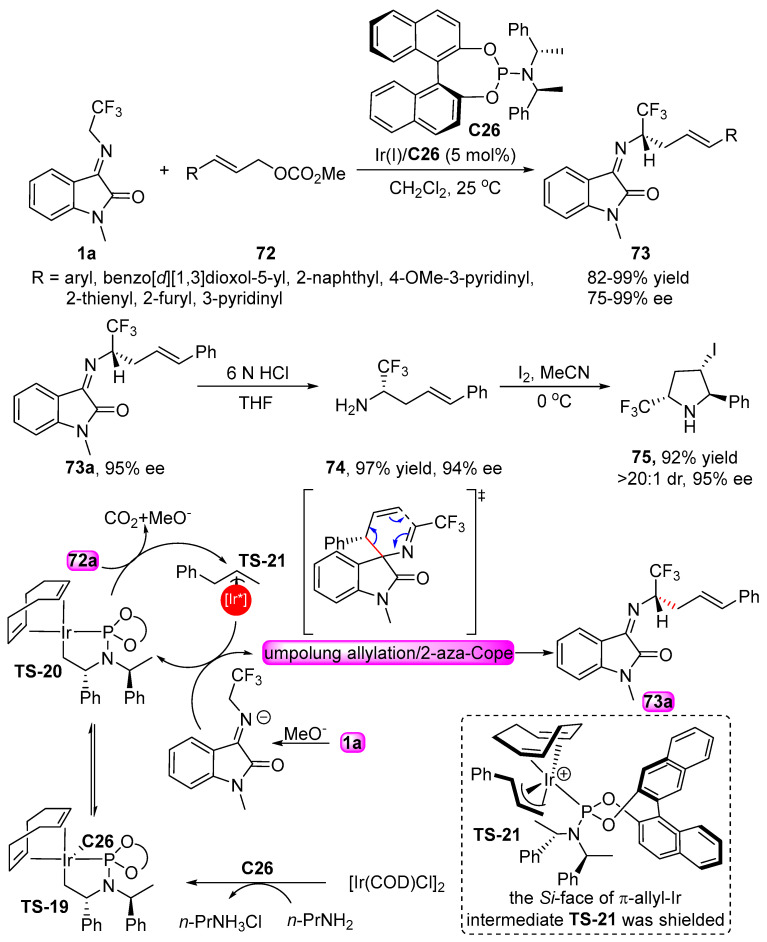
Catalytic asymmetric cascade umpolung allylation/2-aza-Cope rearrangement reactions of *N*-2,2,2-trifluoroethylisatin ketimine.

In 2019, Wang and co-workers described for the first time that trifluoroethylisatin ketimines have the characteristics of polarity reversal and developed a chiral iridium-catalyzed allylation/2-aza-Cope rearrangement cascade reaction between trifluoroethylisatin ketimine **1a** and allylic carbonates **72** (Scheme 26) [43]. This asymmetric catalytic reaction has broad substrate tolerance and provides a new method for the synthesis of α-trifluoromethyl homoallylic amine derivatives **73** with good to excellent enantioselectivities (75–99% ee) in high yields (82–99%). The gram-scale preparation proceeded smoothly under optimal conditions, which further proves the practicability of this synthetic method. The adduct **73a** was hydrolyzed under acidic conditions to obtain its primary amine derivative **74** in 97% yield and 94% ee. Subsequent treatment of primary amines **74** with I_2_ allowed the construction of biologically important trifluoromethylpyrrolidine **75,** containing three stereocenters with exclusive diastereoselectivity at 92% yield. In addition, the researchers proposed a possible transition-state model for the catalytic reaction. First, allylic carbonate **72a** undergoes a coordination reaction with iridacycle **TS-20**, followed by oxidative addition–decarboxylation to generate Ir-π-allyl species **TS-21** and the anion MeO^−^, and the latter serves as the base for the deprotonation of ketimine **1a**. Then, enantioselective umpolung allylation occurs between the substrates to form branched allylation intermediates. The steric congestion caused by the adjacent oxindole ring and phenyl group facilitates a spontaneous 2-aza-Cope rearrangement reaction, ultimately delivering the observed linear α-trifluoromethyl homoallylic amine derivatives.

**Scheme 27:**
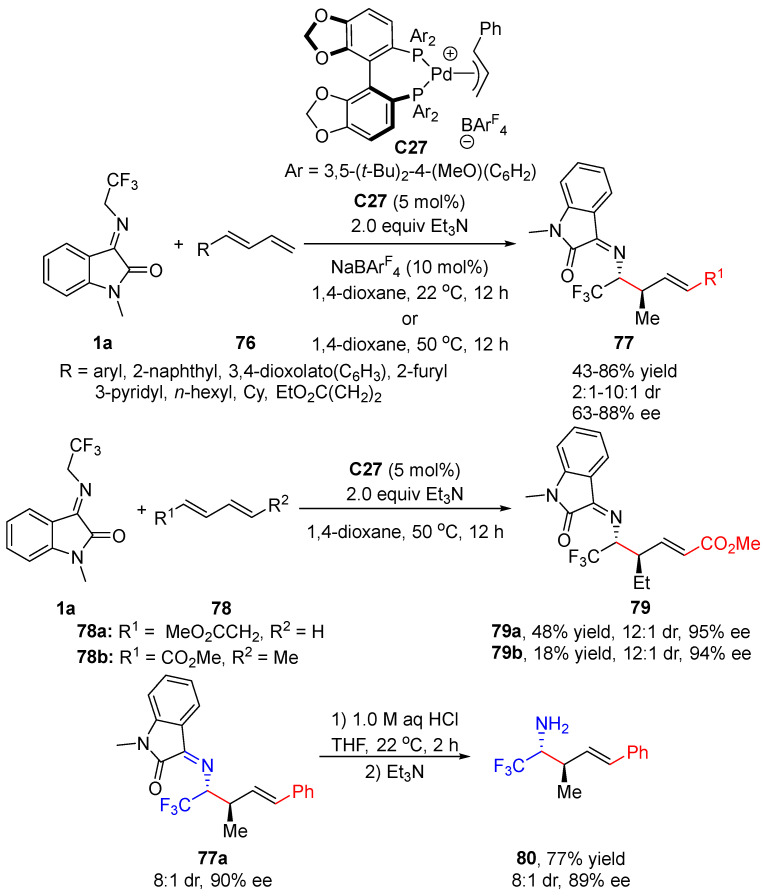
Organocatalyzed γ-selective coupling reaction of trifluoroethylisatin ketimine with terminal olefin of dienes.

A novel palladium-catalyzed highly regioselective asymmetric hydroalkylation reaction between trifluoroethylisatin ketimine **1a** and terminal dienes **76** was presented by the Malcolmson group in 2020 (Scheme 27) [44]. This method was the first to efficiently and stereoselectively synthesize α-trifluoromethyl homoallylamine derivatives using internal olefins and further expands the synthesis strategy of this type of chiral compound. Interestingly, the coupling reaction with **1a** undergoes diene isomerization when using hexadienoate **78a**, providing homoallylamine with an ester-conjugated, ethyl-substituted stereogenic center. However, its internal diene analogues **78b** can also be used to synthesize acrylate with similar stereoselectivity but lower conversion. The gram-scale reaction obtained the target product in 82% yield under optimal conditions while maintaining its stereoselectivity. In addition, the isatin moiety can be removed from the adduct under acidic conditions to obtain a free primary amine derivative **80** with a 77% yield.

**Scheme 28:**
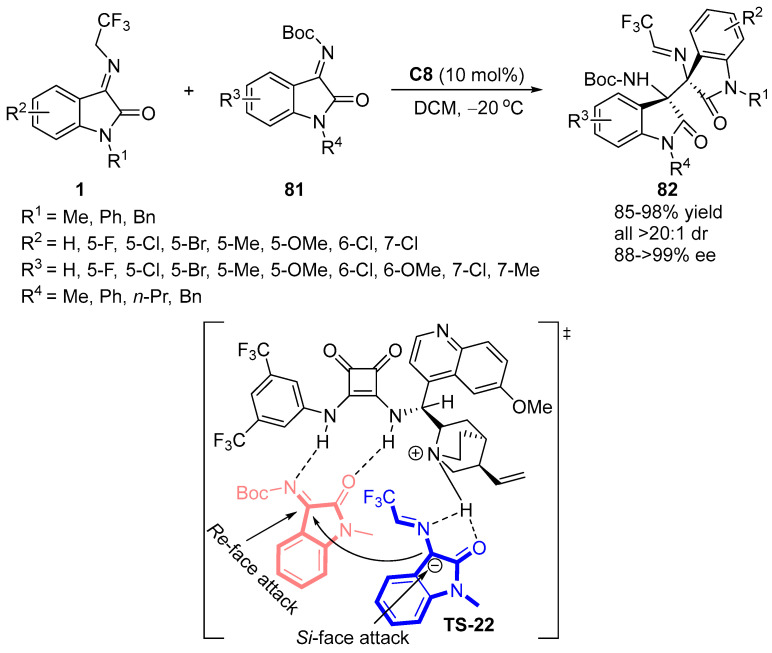
Catalytic asymmetric umpolung cross-Mannich reaction of trifluoroethylisatin ketimines.

In 2020, Lu and co-workers established the cross-Mannich reaction of trifluoroethylisatin ketimines **1** with cyclic ketimines **81** under the catalysis of the chiral bifunctional squaramide catalyst **C8** through a polarity inversion strategy (Scheme 28) [45]. Under the optimal catalytic conditions, chiral vicinal tetrasubstituted diamine derivatives with excellent diastereoselectivities (all >20:1 dr) and enantioselectivities (88–>99% ee) were synthesized in excellent yields (85–98%). This method has the characteristics of wide substrate tolerance, is suitable for gram-scale preparation, and has high chemical/regioselectivity, which provide prerequisites for its practical application in biological activity evaluation research. Furthermore, the researchers proposed a possible catalytic reaction mechanism based on the absolute configuration of the products. The catalyst plays a dual activation role in this reaction (**TS-22**). The trifluoroethylisatin ketimine was partially deprotonated by the tertiary amine of the catalyst and activated by double hydrogen bonds. Meanwhile, the carbonyl and ketimine groups of the isatin-derived *N*-Boc ketimine are immobilized and activated by hydrogen bonds formed by the squaramide moiety of the catalyst. Activated trifluoroethylisatin ketamine attacks the isatin-derived *N*-Boc ketimine from *Re*-face to form the observed product.

**Scheme 29:**
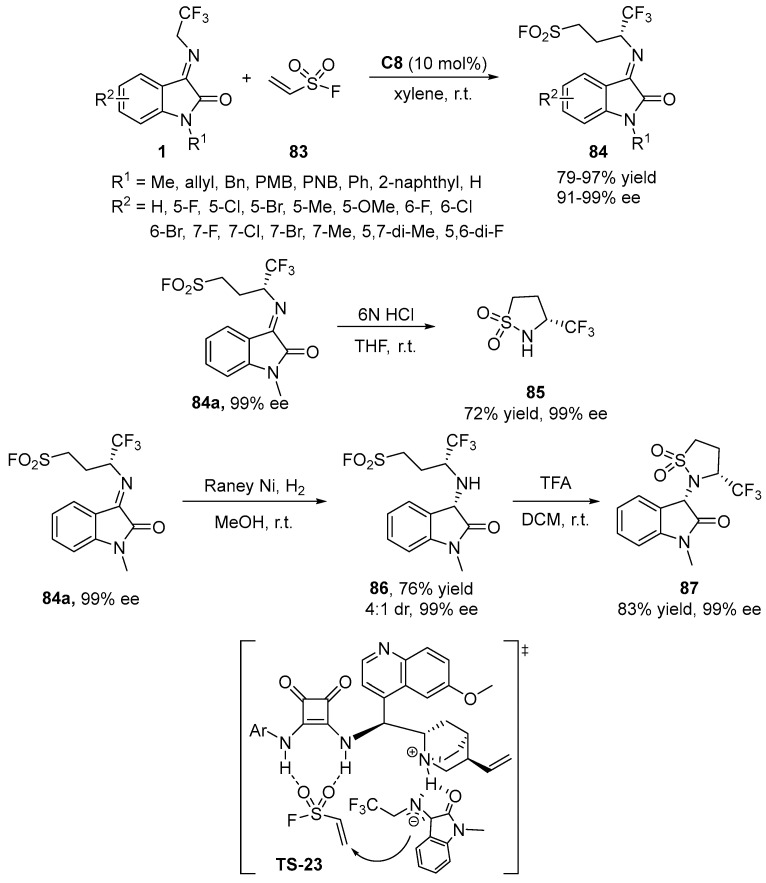
Squaramide-catalysed asymmetric Michael addition between *N*-2,2,2-trifluoroethylisatin ketimines and ethylene sulfonyl fluoride.

A highly efficient asymmetric Michael addition reaction between *N*-2,2,2-trifluoroethylisatin ketimines **1** and ethylene sulfonyl fluoride **83** was realized by the Yan group in 2021 (Scheme 29) [46]. This method utilizes quinine-derived squaramide catalysts **C8** to obtain a series of isatin-derived α-(trifluoromethyl)imine derivatives **84** with diverse structures and excellent enantioselectivities (91–99% ee) in excellent yields (79–97%). The gram-scale reaction proceeded well under optimal conditions, further demonstrating the applicability of the method. In addition, the derivatization experiment on the product was carried out. The product **84a** was hydrolyzed under acidic conditions to obtain a chiral γ-trifluoromethyl-γ-sultam heterocyclic compound **85** with excellent enantioselectivity, which is a useful skeleton in drug research. On the other hand, the adduct **84a** gave the secondary amine derivative **86** with good stereoselectivity and a 76% yield by catalytic hydrogenation. Further treatment of product **86** with TFA allowed intramolecular cyclization to synthesize sultam derivative **87** in 83% yield. Based on the experimental results, the researchers proposed a possible transition state model (**TS-23**).

### 2.2. Catalytic Racemization Reaction of Trifluoroethyl Isatin Ketimines

#### 2.2.1. Catalytic Diastereoselectic [3 + 2] Cycloaddition Reaction

**Scheme 30:**
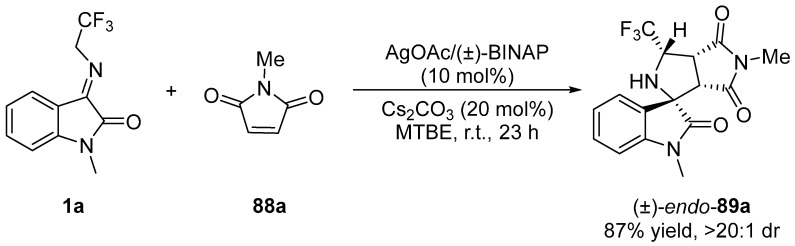
Ag-Catalyzed [3 + 2] cycloaddition reaction of *N*-2,2,2-trifluoroethylisatin ketimine.

In 2016, Carretero and co-workers reported a case of silver-catalyzed [3 + 2] cycloaddition reactions of *N*-2,2,2-trifluoroethylisatin ketimine **1a** with maleimide **88a** (Scheme 30) [47]. By using AgOAc/(±)BINAP as the catalyst system, the desired product **89a** was obtained with 87% yield and >20:1 dr.

**Scheme 31:**
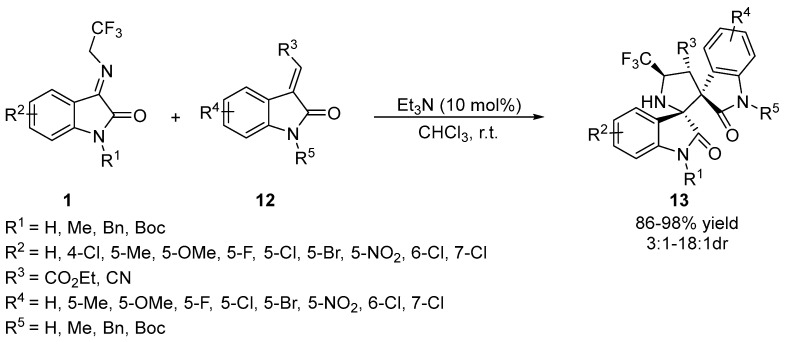
Base-catalyzed diastereoselective [3 + 2] cycloaddition reaction of *N*-2,2,2-trifluoroethylisatin ketimines with methyleneindolinones.

A highly efficient base-catalyzed diastereoselective [3 + 2] cycloaddition reaction of *N*-2,2,2-trifluoroethylisatin ketimines **1** with methyleneindolinones **12** was developed by the Lin group in 2017 (Scheme 31) [48]. The reaction has broad substrate tolerance under mild conditions, and CF_3_-containing 3,3′-pyrrolidinyl-dispirooxindole derivatives **13** with good diastereoselectivities (3:1–18:1 dr) can be obtained in excellent yields (86–98%).

**Scheme 32:**
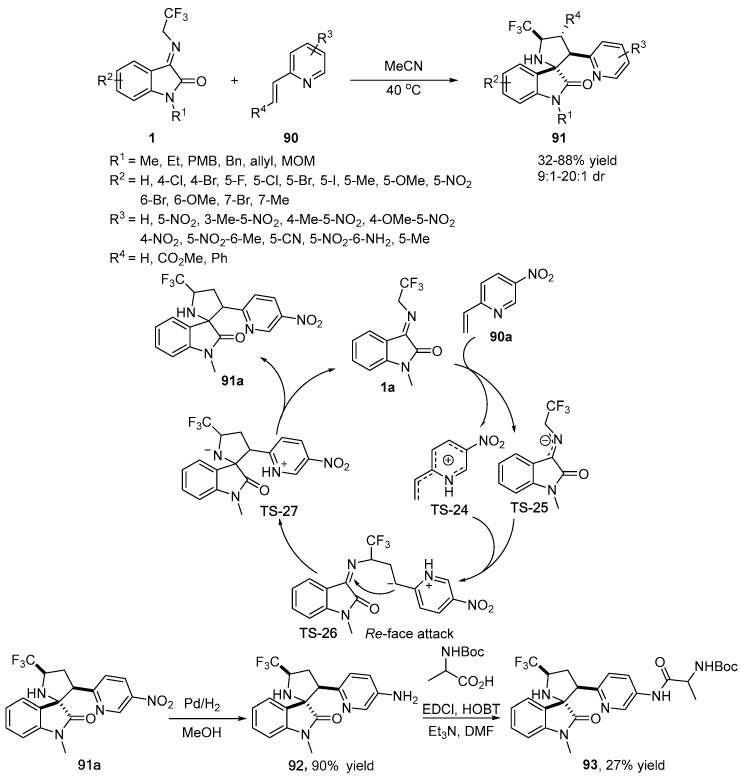
Catalyst-free self-catalyzed [3 + 2] cycloaddition reaction of *N*-2,2,2-trifluoroethylisatin ketimines with vinylpyridines.

In 2018, Shi and co-workers disclosed a catalyst-free, self-catalyzed [3 + 2] cycloaddition reaction of trifluoroethylisatin ketimines **1** with vinylpyridines **90** (Scheme 32) [49]. This reaction provides a facile and feasible method for the construction of a series of CF_3_-containing spiropyrrolidin-3,2′-oxindole derivatives **91** with moderate to good yields (32–88%) and good diastereoselectivities (9:1–20:1 dr). The researchers studied the reaction mechanism through control experiments, DFT calculations of p*Ka* values, and kinetic curves, revealing that this reaction is completed by mutual activation between the substrates. First, substrate **1a** is deprotonated by vinylpyridine **90a** to produce protonated vinylpyridine **TS-24** and intermediate **TS-25**. Then the intermolecular Michael addition reaction occurs to produce the intermediate **TS-26**. The intermediate **TS-27** was obtained by the intramolecular Mannich reaction of carbanion attacking the imine moiety of **1b** from *Re*-face. Finally, the intermediate **TS-27** undergoes intramolecular proton transfer to produce the required product **91a**. In order to increase the practicability of the product, a derivatization experiment was carried out on it. Treating the adduct with Pd/H_2_ in methanol can reduce its nitro group to an amino group with a yield of 90%. Subsequently, the product **92** was condensed with amino acids, and a derivative **93** with potential application value in biomolecular synthesis was constructed at a 27% yield.

**Scheme 33:**
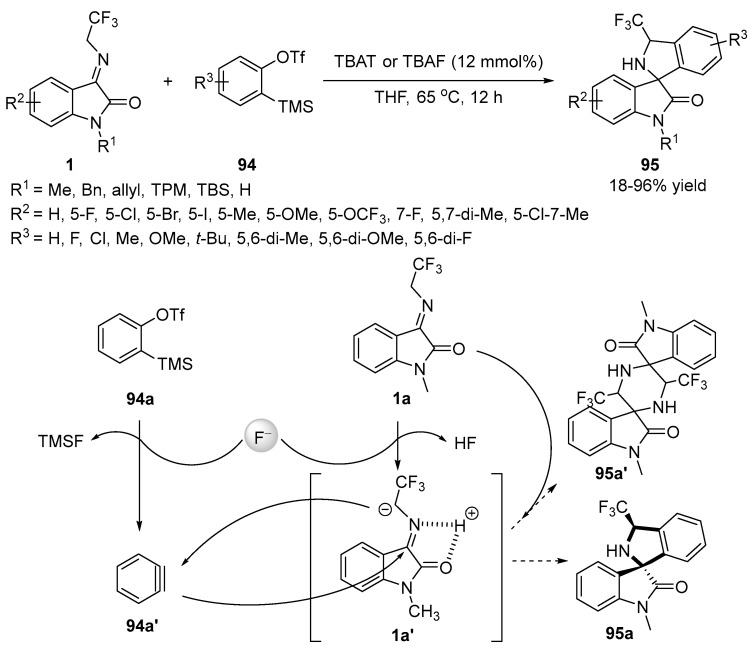
Catalytic diastereoselective [3 + 2] cycloaddition reaction of *N*-2,2,2-trifluoroethylisatin ketimines with benzynes.

A novel strategy for the [3 + 2] cycloaddition of *N*-2,2,2-trifluoroethylisatin ketimines **1** to benzynes **94** was described by the Ko group in 2018 (Scheme 33) [50]. This reaction proceeds smoothly in the presence of a weak base such as TBAF or TBAT, and spiro[oxindole-3,2′-pyrrolidine] derivatives **95** can be constructed in good yields (32–88%). Furthermore, the researchers proposed a possible catalytic mechanism based on the experimental results. When the imine **1a** is treated with a weak base such as fluoride, it can be deprotonated and transformed into the azomethine ylide **1a′**. Subsequently, the intermediate **1a′** of azomethine ylide reacted with the in-situ generated benzyne **94a′** to form the desired product by the [3 + 2] cycloaddition reaction. However, two molecules of the azomethine ylide intermediate **1a′** can be transformed into a dimer **95a′** by a [3 + 3] cycloaddition reaction in the presence of TBAF.

**Scheme 34:**
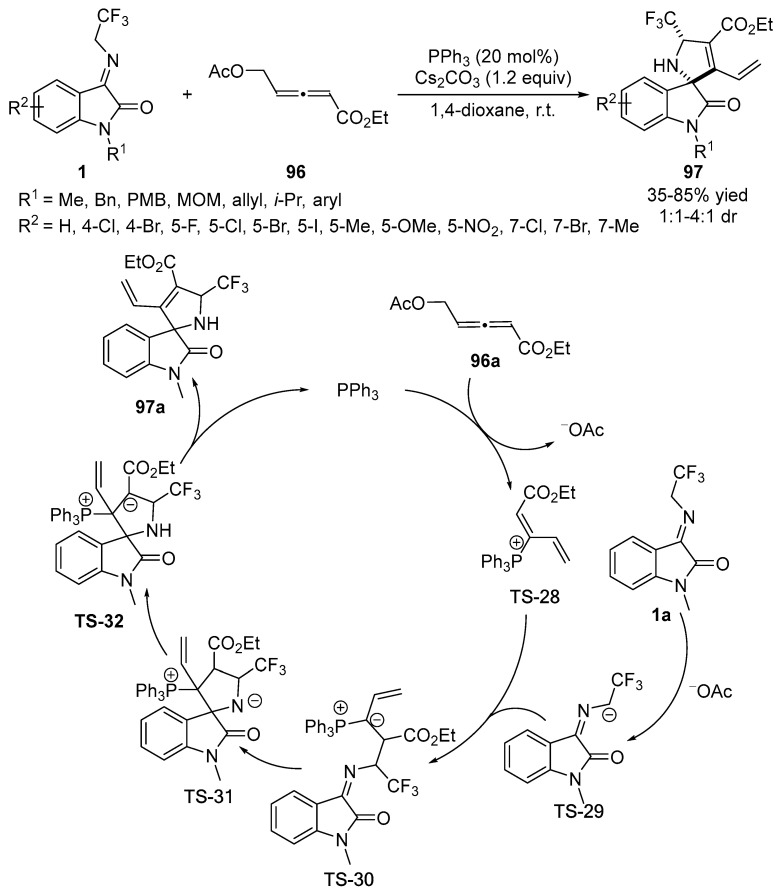
Phosphine-catalyzed [3 + 2] annulation of *N*-2,2,2-trifluoroethylisatin ketimines with γ-substituted allenoates.

In 2019, Shi and co-workers presented a phosphine-catalyzed [3 + 2] cycloaddition reaction of *N*-2,2,2-trifluoroethylisatin ketimines **1** with γ-substituted allenoates **96** (Scheme 34) [51]. This reaction can construct spiro[indoline-3,2′-pyrrole] skeleton compounds **97** in moderate to good yields (35–85%) with acceptable diastereoselectivities (1:1–4:1 dr) in the presence of triphenylphosphine. In addition, researchers proposed a possible mechanism for this novel [3 + 2] cycloaddition reaction catalyzed by phosphine. First, the phosphine catalyst attacks the β-position of allenoates **96a** to eliminate the acetate group to generate intermediate **TS-28**. Then, substrate **1a** was deprotonated by the generated ^−^OAc to obtain intermediate **TS-29**, which further underwent an intermolecular Michael addition reaction with intermediate **TS-28** to generate intermediate **TS-30**. Subsequently, the intermediate undergoes an intramolecular Mannich reaction to form intermediate **TS-31**, which is transferred by protonation to obtain intermediate **TS-32**. Finally, the desired product is produced, and the catalyst is regenerated, completing the catalytic cycle.

**Scheme 35:**
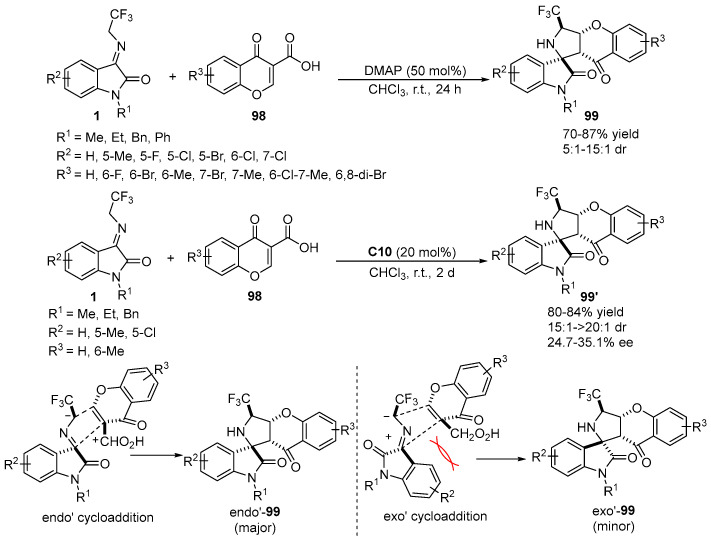
DMAP-catalyzed decarboxylative [3 + 2] annulation of *N*-2,2,2-trifluoroethylisatin ketimines with 3-carboxylic acid chromones.

An efficient DMAP-catalyzed decarboxylative [3 + 2] annulation of *N*-2,2,2-trifluoroethylisatin ketimines **1** with 3-carboxylic acid chromones **98** was established by the Zhou group in 2020 (Scheme 35) [52]. A series of trifluoromethylated chromanone-fused pyrrolidinyl spirooxindoles **99** with high diastereoselectivities (5:1–15:1 dr) and potential bioactivity were synthesized in good yields (70–87%). In addition, the researchers also preliminarily attempted the asymmetric decarboxylation [3 + 2] cycloaddition reaction catalyzed by chiral bifunctional squaramide catalysts. However, preliminary experimental results are not satisfactory. According to the absolute configuration of the product, the researchers believe that the cycloaddition reaction mainly proceeds through the endo’-transition state.

**Scheme 36:**
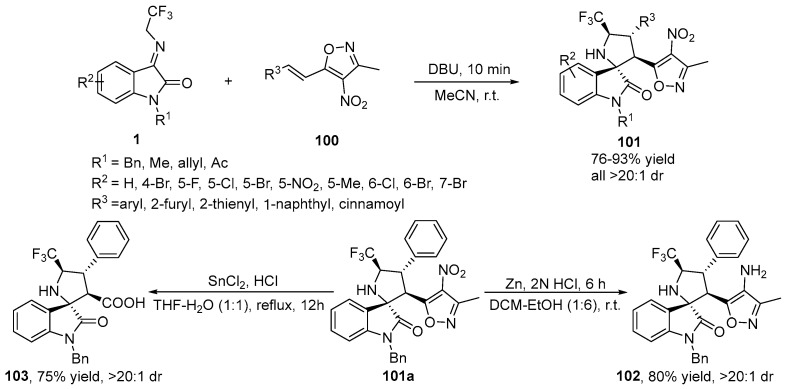
Highly diastereoselective [3 + 2] cycloaddition of *N*-2,2,2-trifluoroethylisatin ketimines with styrylisoxazoles.

In 2021, Huang and co-workers realized a highly diastereoselective [3 + 2] cycloaddition of *N*-2,2,2-trifluoroethylisatin ketimines **1** with styrylisoxa-zoles **100** (Scheme 36) [53]. A series of CF_3_-containing 3′-(nitroisoxazol)spiro [pyrrolidin-3,2′-oxindole] derivatives **101** with excellent diastereoselectivities (all >20:1 dr) were obtained in good to excellent yields (76–93%) under mild conditions. In addition, the comprehensive practicality of this catalytic reaction is demonstrated by gram-scale preparation and derivatization experiments. Treatment of the adduct with Zn and HCl in DCM/EtOH (1:6) could reduce it to the amino derivative **102** with an 80% yield. When the adduct was treated with HCl and SnCl_2_ in THF/H_2_O (1:1), the ring-opened product **103** could be obtained with a 75% yield. Either strategy does not affect its diastereoselectivity.

Subsequently, Han’s group synthesized a series of nitroisoxazole-containing spiro[pyrrolidin-oxindole] derivatives **101** in the same way [54]. The difference is that the researchers evaluated its pharmacological activity as a glutathione peroxidase 4 (GPX4)/mouse double minute 2 (MDM2) dual inhibitor and found that the resulting compound exhibited strong activity against both targets. Through in-depth experimental research, compounds with the same activity in vitro and in vivo were selected.

**Scheme 37:**
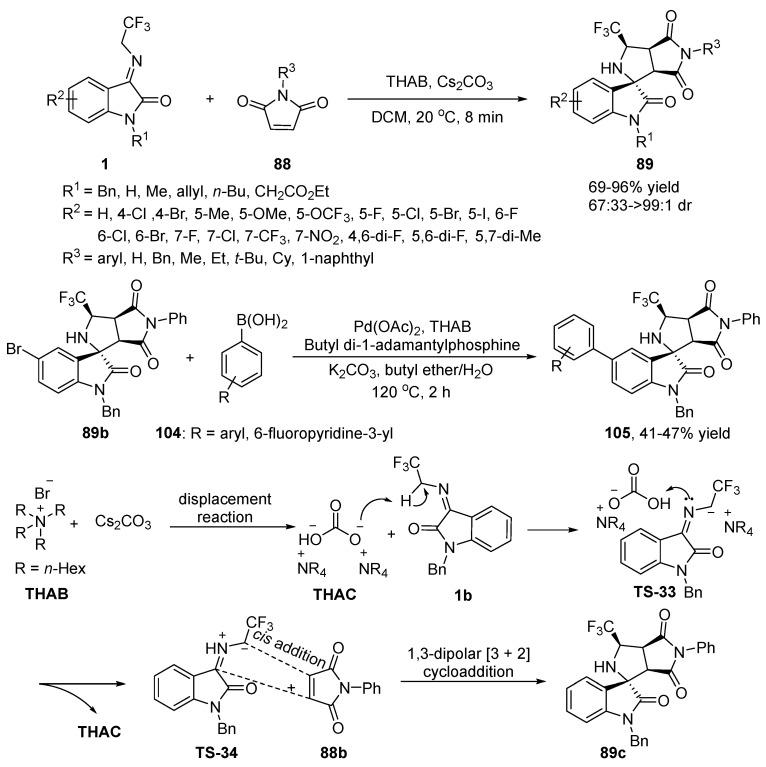
PTC-catalyzed diastereoselective [3 + 2] cycloaddition reaction of *N*-2,2,2-trifluoroethylisatin ketimines with maleimides.

A highly efficient and practical diastereoselective [3 + 2] cycloaddition reaction of *N*-2,2,2-trifluoroethylisatin ketimines **1** with maleimides **88** was reported by the Chen group in 2021 (Scheme 37) [55]. This reaction affords a series of complex trifluoromethyl spiro-fused[succinimide-pyrrolidine-oxindole] derivatives **89** with good diastereoselectivities (67:33–>99:1 dr) in moderate to excellent yields (69–96%) in the presence of a phase transfer catalyst. The gram-scale preparation and derivatization experiments demonstrated the application prospects of this synthetic strategy. The Suzuki–Miyaura cross-coupling reaction between the adduct and arylboronic acid was carried out under optimized conditions, and the target product **105** was obtained in moderate yields. In addition, the researchers proposed a possible reaction mechanism based on the absolute configuration of the product and previous studies. Initially, THAB undergoes a displacement reaction with Cs_2_CO_3_ on its solid surface to produce THAC. The trifluoroethyl imine **1b** is then deprotonated by the carbonate anion of THAC and transferred into the DCM liquid phase. Subsequently, the imine moiety of the intermediate **TS-33** captures the proton of tetrahexylammonium bicarbonate to generate the reactive azomethine ylide **TS-34** and release THAC. Finally, yelide **TS-34** and maleimide **88b** undergo cycloaddition conversion to deliver the desired product.

**Scheme 38:**
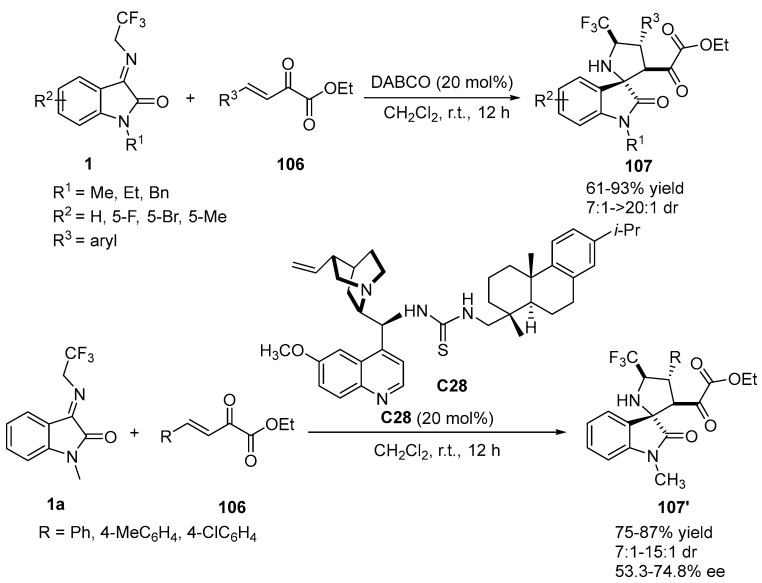
Highly diastereoselective [3 + 2] cycloaddition reaction of *N*-2,2,2-trifluoroethylisatin ketimines with β,γ-unsaturated α-keto esters.

In 2021, Wang and co-workers developed an efficient diastereoselective [3 + 2] cycloaddition of *N*-2,2,2-trifluoroethylisatin ketimines **1** with, β,γ-unsaturated α-keto esters **106** in the presence of the catalyst DABCO (Scheme 38) [56]. This strategy enables the construction of CF_3_-containing spiro[pyrrolidin-3,2′-oxindole] derivatives **107** with good diastereoselectivities (7:1–>20:1 dr) and moderate-to-excellent yields (61–93%) under optimal conditions. Subsequently, the researchers conducted a preliminary exploration of this asymmetric catalytic reaction using a bifunctional thiourea catalyst. In addition, the researchers screened the biological activity of these compounds on K562 leukemia cells by the MTT method.

**Scheme 39:**
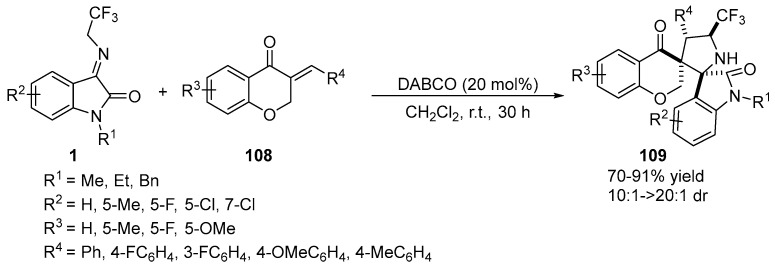
Diastereoselective domino [3 + 2] cycloaddition reaction of *N*-2,2,2-trifluoroethylisatin ketimines with benzylidenechromanones.

A new strategy for the synthesis of trifluoromethyl bispiro-[oxindole-pyrrolidine-chromanone] derivatives with diverse structures was disclosed by the Tian group in 2021 (Scheme 39) [57]. The method uses DABCO to catalyze the diastereoselective [3 + 2] cycloaddition reaction of trifluoroethylisatin ketimines **1** and benzylidenechromanones **108** and obtains the target products **109** with good diastereoselectivities (10:1–>20:1 dr) in high yields (70–91%). The researchers also preliminarily explored the catalytic effect of chiral organocatalysts in this reaction. However, the selected catalysts cannot achieve satisfactory results. In addition, the biological activity of the selected compounds was tested by the MTT method.

**Scheme 40:**
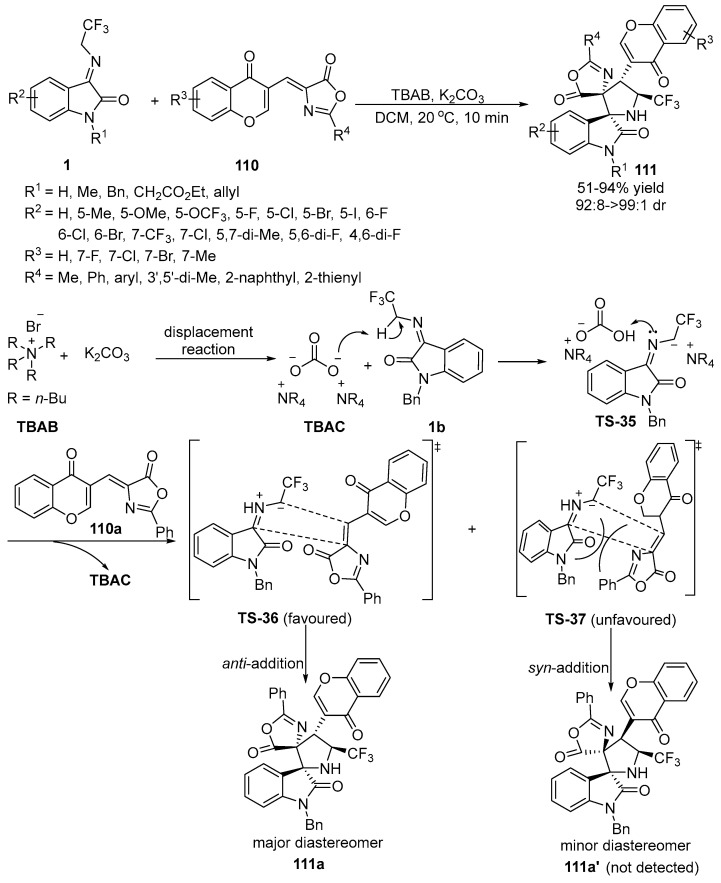
PTC-catalyzed diastereoselective [3 + 2] cycloaddition reaction of *N*-2,2,2-trifluoroethylisatin ketimines with (*Z*)-4-((chromone-3-yl)methylene)oxazolones.

In 2021, Chen and co-workers described a new method for the rapid construction of bispiro heterocycles with five pharmacophores by using phase transfer catalysis (Scheme 40) [58]. Under mild conditions, the diastereoselective [3 + 2] cycloaddition reaction of *N*-2,2,2-trifluoroethylisatin ketimines **1** with (*Z*)-4-((chromone-3-yl)methylene)oxazolones **110** took place, and the desired product with excellent diastereoselectivities (92:8–>99:1 dr) was produced in moderate to excellent yields (51–94%). The kinetic control mechanism of the catalytic reaction was explored through different catalytic systems. In addition, the researchers also discussed the catalytic mechanism of PTC in depth, but since it has been described in Scheme 37, it will not be repeated here.

**Scheme 41:**
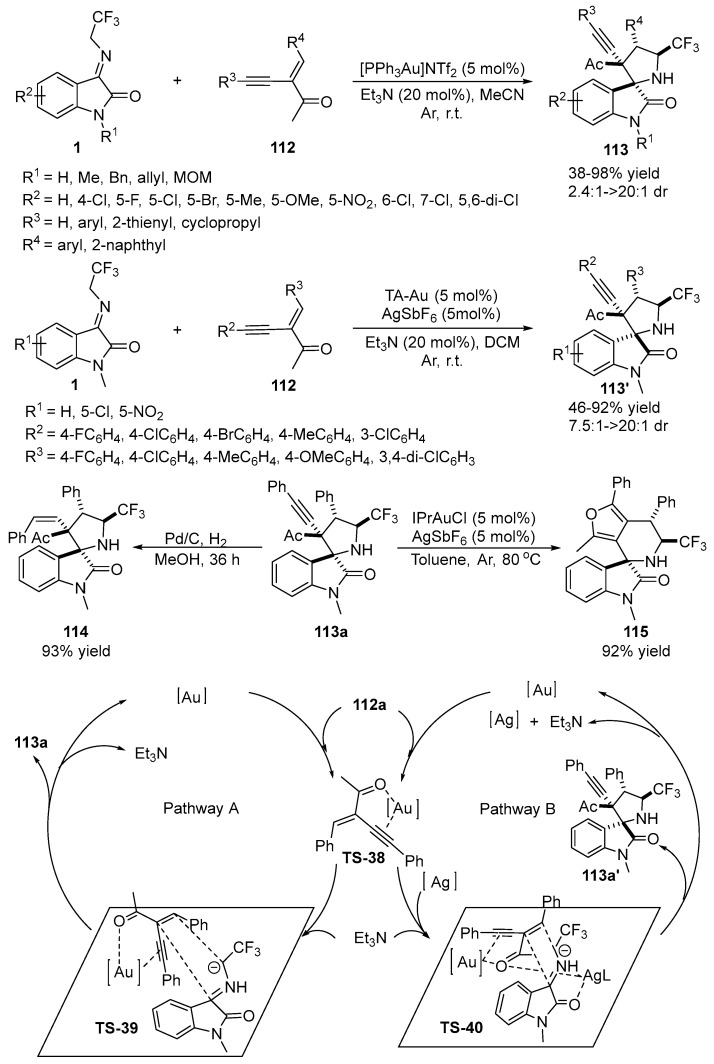
Diastereoselectivity-switchable gold-catalyzed formal [3 + 2] cycloaddition reaction of *N*-2,2,2-trifluoroethylisatin ketimines with yne enones.

The unexpected gold-catalyzed diastereoselective [3 + 2] cycloaddition reaction of *N*-2,2,2-trifluoroethylisatin ketimines **1** with yne enones **112** was presented by the Su group in 2021 (Scheme 41) [59]. The catalytic reaction can obtain diastereoisomers (**113** or **113′**) with good diastereoselectivities in moderate to excellent yields under different catalytic systems. The practicability of the method was further demonstrated by gram-scale preparation and derivatization experiments. Treatment of adduct **113a** with Pd/C and H_2_ in methanol afforded the alkene derivative **114** in a 93% yield. More interestingly, in the presence of IPrAuCl/AgSbF_6_, cycloadduct **113a** can undergo 1,2′-alkyl migration at a relatively higher temperature to generate furan-fused spiroindole **115**. In addition, the researchers proposed the catalytic mechanism of the reaction based on the experimental results and previous studies. The reaction synthesized its diastereoisomers separately through two different pathways.

**Scheme 42:**
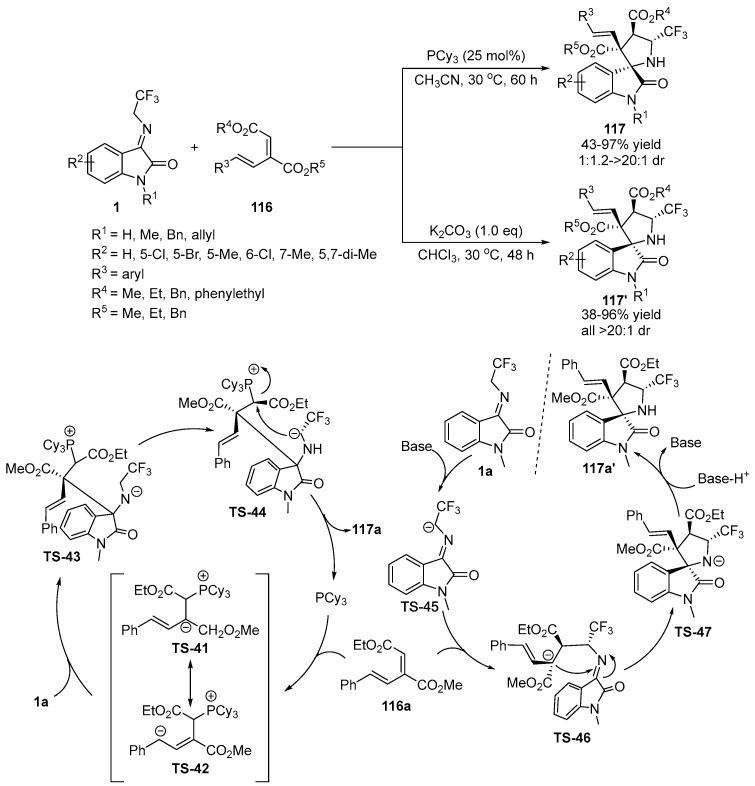
Base-promoted diastereoselective [3 + 2] cycloaddition reaction of *N*-2,2,2-trifluoroethylisatin ketimines with conjugated dienes.

In 2022, Duan and co-workers established a new method for the diastereoselective construction of fully disubstituted spiro[indoline-3,2′-pyrrolidin]-2-one derivatives through base-promoted [3 + 2] cycloaddition reactions (Scheme 42) [60]. Interestingly, different configurations of products were obtained when different bases were used as catalysts. Whether Lewis base (PCy_3_) or Brøwns base (K_2_CO_3_) was used as the catalyst, both of them could obtain spiroheterocyclic derivatives (**117** or **117′**) with excellent diastereoselectivity in good yields. The difference is that the products obtained by the two catalytic reactions are diastereoisomers. Based on the experimental results and previous studies, two plausible catalytic mechanisms were proposed. When PCy_3_ was used as the catalyst, the phosphine catalyst first underwent nucleophilic addition with conjugated diene **116a** to obtain the zwitterionic intermediate **TS-41**. Then intermediate **TS-41** attacks the trifluoroethylisatin ketimine **1a** from a specific direction to undergo a nucleophilic addition reaction to generate intermediate **TS-43**. Finally, intermediate **TS-44** undergoes the S_N_2 substitution reaction to form the desired product *endo*-**117a** and regenerates the phosphine catalyst. However, when K_2_CO_3_ was used as the Brønsted base, substrate **1a** was deprotonated to form the intermediate **TS-45**. Then an intermolecular Michael addition reaction with conjugated dienes was carried out to afford intermediate **TS-46**. Subsequently, the intramolecular Mannich reaction and protonation reaction occurred to produce the corresponding product, *exo’*-**117a,** and regenerate the catalyst.

#### 2.2.2. Catalytic Diastereoselectic [3 + 3] Cycloaddition Reaction

**Scheme 43:**
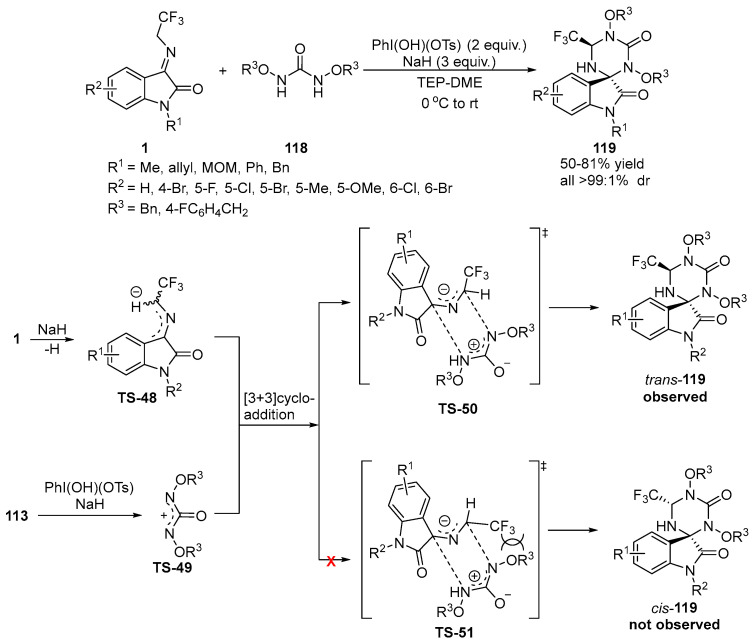
Diastereoselective formal [3 + 3] cycloaddition reaction of *N*-2,2,2-trifluoroethylisatin ketimines with *N,N′*-dialkyloxyureas.

A highly efficient diastereoselective [3 + 3] cycloaddition reaction of *N*-2,2,2-trifluoroethylisatin ketimines **1** with *N,N′*-dialkyloxyureas **118** was realized by the Zhao group in 2019 (Scheme 43) [61]. This reaction has a broad substrate scope and enables the synthesis of spiro-1,3,5-triazinan-2-one derivatives **119** with excellent diastereoselectivities (all >99:1 dr) in moderate to good yields (50–81%). Furthermore, the researchers proposed a possible catalytic mechanism based on the experimental results to elucidate the reaction. First, substrate **1** was deprotonated to form its ylide, **TS-48**, while substrate **118** was oxidized to afford its diaza-allyl cation, **TS-49**. The in situ-generated intermediate undergoes a [3 + 3] cycloaddition via two possible transition states to generate the product. However, there is a strong steric repulsion between the CF_3_ and OR^3^ groups in the transition state **TS-51**. Therefore, **TS-50** was thermodynamically more stable and was dominated by the formation of *trans*-**119**.

**Scheme 44:**
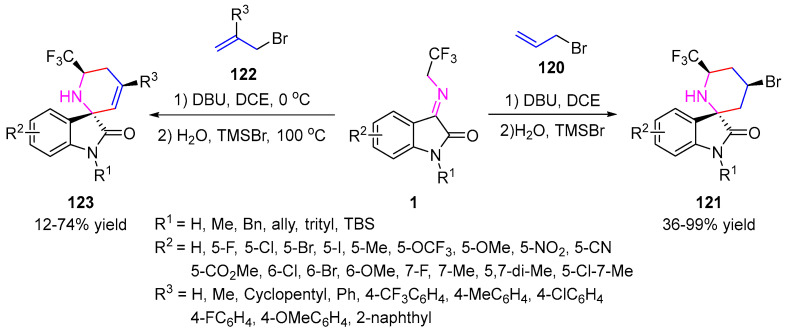
Organocatalytic [3 + 3] cycloaddition reaction of *N*-2,2,2-trifluoroethylisatin ketimines with allyl bromides.

In 2021, Ko and co-workers reported a one-pot process of umpolung allylation/aza-Prins cyclization of *N*-2,2,2-trifluoroethylisatin ketimines **1** with allyl bromide (Scheme 44) [62]. This reaction enables the reaction with allyl bromide **120** at lower temperatures to synthesize spiro[indoline-3,2′-piperidin]-2-one derivatives **121** in moderate to excellent yields (36–99%). However, this reaction with a substituted allyl bromide **122** enables the synthesis of 5′,6′-dihydro-1′*H*-spiro[indoline-3,2′-pyridin]-2-one derivatives **123** in moderate yields (12–74%) at elevated temperatures. This reaction is the first reported aza-Prins cyclization, taking advantage of the umpolung property of *N*-2,2,2-trifluoroethylisatin ketimines.

Remarkably, this study was not compatible with TMSX (X=Cl, I, etc.) in the one-pot method. To solve this problem, Ko’s group also developed a step-by-step process based on this study in the same year that overcame the limitations of the one-pot method and successfully performed the [3 + 3] aza-Prins cyclization reaction with TMSX (X=Cl, I, etc.) [63].

#### 2.2.3. Catalytic Diastereoselectic [3 + 5] Cycloaddition Reaction

**Scheme 45:**
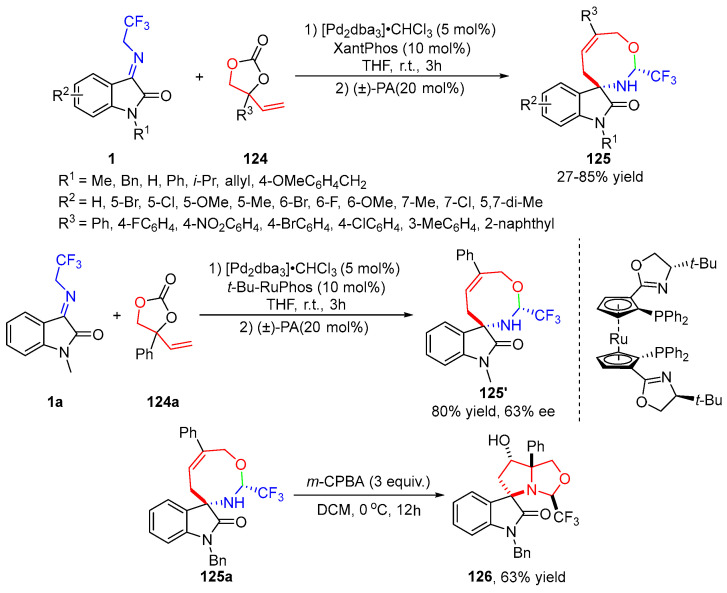
Palladium-catalyzed diastereoselective formal [3 + 5] cycloaddition reaction of *N*-2,2,2-trifluoroethylisatin ketimines with VECs.

An efficient Pd-catalyzed diastereoselective formal [3 + 5] cycloaddition of *N*-2,2,2-trifluoroethylisatin ketimines **1** with aryl substituted vinylethylene carbonates (VECs) **124** was disclosed by the Shi group in 2019 (Scheme 45) [64]. This reaction exhibits good substrate tolerance in the presence of Brønsted acid and constructs CF_3_-containing spirooxindole derivatives fused with an eight-membered ring in high yields (27–85%). The researchers tried using *t*-Bu-RuPhos as a chiral phosphine ligand, and the asymmetric reaction synthesized a chiral spiro compound **125′** with 63% ee in 80% yield. In addition, the adduct **125a** treated with *m*-chloroperoxybenzoic acid (*m*-CPBA) in DCM can be converted into the spiroxyindole derivative **126** with a 63% yield.

Subsequently, Zhao and co-workers also presented a diastereoselective [3 + 5] cycloaddition reaction of *N*-2,2,2-trifluoroethylisatin ketimines **1** with VECs **119** [65]. Differently from before, the reaction was carried out under the catalyst system of Pd(PPh_3_)_4_, PPh_3_, and pyridine. However, this catalytic system enables the synthesis of the desired product with excellent diastereoselectivities (all >20:1 dr) and good to excellent yields (75–97%). 

**Scheme 46:**
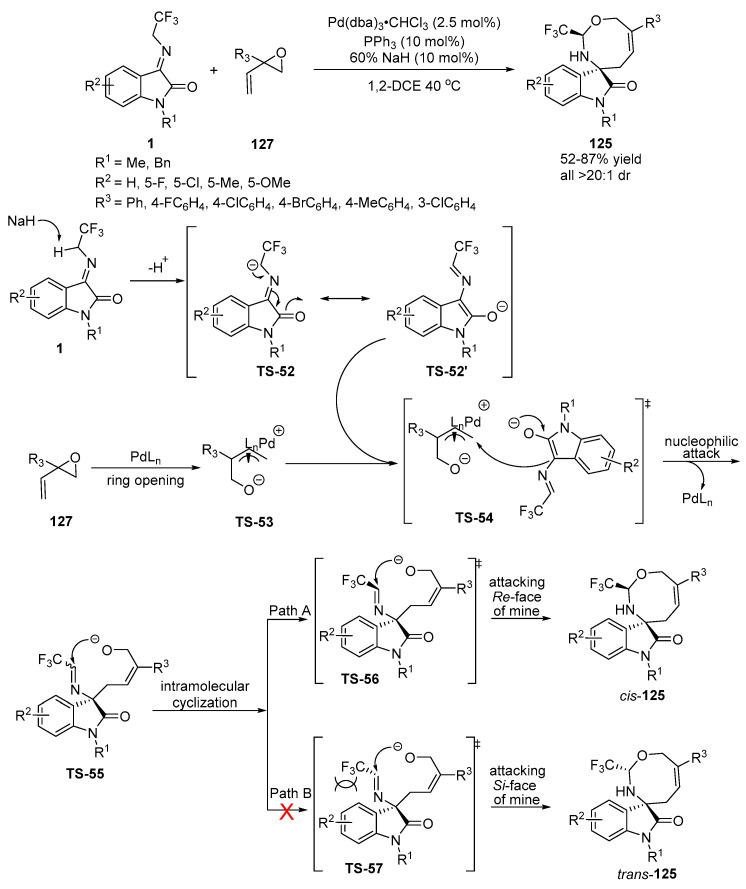
Palladium-catalyzed diastereoselective formal [3 + 5] cycloaddition reaction of *N*-2,2,2-trifluoroethylisatin ketimines with vinyloxiranes.

A similar diastereoselective [3 + 5] cycloaddition reaction of trifluoroethylisatin ketimines **1** with vinyloxiranes **127** was established by the Zhou group in 2021 (Scheme 46) [66]. In the presence of Pd_2_(dba)_3_·CHCl_3_, PPh_3,_ and 60% NaH, this reaction enables the synthesis of medium-heterocycle-fused spirooxindole compounds with excellent diastereoselectivities (all >20:1 dr) in moderate to good yields (52–87%). To explain the formation of the *cis*-products, the researchers proposed a possible catalytic reaction mechanism. First of all, the imine is deprotonated by NaH to provide the azomethine ylide **TS-52**, which readily resonates into its enolate **TS-52′**. At the same time, the ring-opening reaction of vinyloxirane **127** under the catalysis of the in situ-formed PdL_n_ complex produces the Pd-π-allyl complex **TS-53**. Then, the generated enolate **TS-52′** attacks the zwitterionic Pd-π-allyl complex **TS-53** via transition state **TS-54** to provide intermediate **TS-55**. At this time, there are two possible pathways for the intermediate to deliver the cyclization products. However, due to the strong repulsion between CF_3_ and benzene groups in the transition state **TS-57**, the transition state **TS-56** is thermodynamically more stable, so the formation of *cis*-products dominates.

## 3. Conclusions

In summary, *N*-2,2,2-trifluoroethylisatin ketimines have been widely used in organocatalytic reactions as a 1,3-dipole, nucleophile, and synthetic “building block” containing trifluoromethyl groups with excellent activity. In order to facilitate readers’ understanding, we classify them according to the two major parts of asymmetric catalysis and diastereoselective synthesis and refine them according to their reaction types. So far, researchers have mainly focused on the application of trifluoroethylketoimine in the [3 + 2] cycloaddition reaction, while the studies of [3 + 3], [3 + 4], and [3 + 5] cycloaddition are relatively limited. In particular, the application of developed synthetic methodologies to the synthesis of active drug molecules or complex natural products has rarely been reported. With the deepening of organic synthesis research, further exploration and expansion of trifluoroethylisatin ketimines in the construction of active drug molecules or their skeletons under the action of various types of organic catalysts and even its application in the synthesis of natural product molecules will become hot and challenging research topics in the future. We believe that in the near future, more and more unexpected organic synthesis methods involving trifluoroethylisatin ketimines will be established and applied to the construction of some important pharmaceutical skeletons.

## Data Availability

Not applicable.
